# Potential Hepatoprotective Effects of *Chamaecyparis lawsoniana* against Methotrexate-Induced Liver Injury: Integrated Phytochemical Profiling, Target Network Analysis, and Experimental Validation

**DOI:** 10.3390/antiox12122118

**Published:** 2023-12-14

**Authors:** Eman Fikry, Raha Orfali, Shaimaa S. El-Sayed, Shagufta Perveen, Safina Ghafar, Azza M. El-Shafae, Maher M. El-Domiaty, Nora Tawfeek

**Affiliations:** 1 Department of Pharmacognosy, Faculty of Pharmacy, Zagazig University, Zagazig 44519, Egypt; efhassan@zu.edu.eg (E.F.); amelshafaey@pharmacy.zu.edu.eg (A.M.E.-S.); noratawfeek@zu.edu.eg (N.T.); 2 Department of Pharmacognosy, College of Pharmacy, King Saud University, P.O. Box 2457, Riyadh 11451, Saudi Arabia; sghafar.c@ksu.edu.sa; 3 Department of Pharmacology and Toxicology, Faculty of Pharmacy, Zagazig University, Zagazig 44519, Egypt; ssothman@pharmacy.zu.edu.eg; 4 Department of Chemistry, School of Computer, Mathematical and Natural Sciences, Morgan State University, Baltimore, MD 21251, USA; shagufta.perveen@morgan.edu

**Keywords:** *Chamaecyparis lawsoniana*, UPLC-ESI-MS/MS, acute liver injury, network pharmacology, docking

## Abstract

Methotrexate (MTX) therapy encounters significant limitations due to the significant concern of drug-induced liver injury (DILI), which poses a significant challenge to its usage. To mitigate the deleterious effects of MTX on hepatic function, researchers have explored plant sources to discover potential hepatoprotective agents. This study investigated the hepatoprotective effects of the ethanolic extract derived from the aerial parts of *Chamaecyparis lawsoniana* (CLAE) against DILI, specifically focusing on MTX-induced hepatotoxicity. UPLC-ESI-MS/MS was used to identify 61 compounds in CLAE, with 31 potential bioactive compounds determined through pharmacokinetic analysis. Network pharmacology analysis revealed 195 potential DILI targets for the bioactive compounds, including TP53, IL6, TNF, HSP90AA1, EGFR, IL1B, BCL2, and CASP3 as top targets. In vivo experiments conducted on rats with acute MTX-hepatotoxicity revealed that administering CLAE orally at 200 and 400 mg/kg/day for ten days dose-dependently improved liver function, attenuated hepatic oxidative stress, inflammation, and apoptosis, and reversed the disarrayed hepatic histological features induced by MTX. In general, the findings of the present study provide evidence in favor of the hepatoprotective capabilities of CLAE in DILI, thereby justifying the need for additional preclinical and clinical investigations.

## 1. Introduction

The liver, being the primary organ responsible for metabolism, plays a crucial role in various physiological processes such as storing liver sugar, synthesizing secretory proteins, and detoxifying harmful substances. Any dysfunction or injury to the liver can lead to adverse effects on the body, and in severe cases, it can even result in death. Consequently, liver-related issues have become a significant concern in public health. One of the common problems associated with liver function is drug-induced liver injury (DILI), which refers to the side effects caused by medications and is often the leading cause of acute liver failure. This condition can not only impede therapeutic progress but also restrict drug development and result in the discontinuation of specific medications from the market [1,2].

Methotrexate (MTX), also known as amethopterin, is a versatile medication that has been proven effective in treating a wide range of medical conditions. It is commonly prescribed for skin disorders such as psoriasis and refractory atopic dermatitis, as well as inflammatory and autoimmune diseases like rheumatoid arthritis, vasculitis, and Crohn’s disease. In addition, it is also used to treat various malignant disorders such as leukemia, lung, breast, and uterine cancers, as well as ectopic pregnancy [3,4,5,6].

Despite its effectiveness, methotrexate has a high efficacy/toxicity ratio, which can lead to multiorgan toxicities due to its lack of selective cytotoxicity [7]. This has raised concerns about its use, particularly in high doses or long-term treatments. Liver-related adverse effects are among the most important complications associated with methotrexate, with liver abnormalities ranging from asymptomatic elevations in liver enzymes to fibrosis and even fatal hepatic necrosis [8]. Oxidative stress is undeniably a significant factor in the development of methotrexate-related abnormalities and its cytotoxic effects [9,10,11,12]. The excessive production of reactive oxygen species (ROS) during methotrexate therapy can impair the antioxidant capacity of the liver and cause damage to cell membranes through lipid peroxidation. This ultimately leads to tissue damage [13,14,15]. Additionally, apoptosis, which is a crucial process for maintaining cellular homeostasis, becomes overactivated in adverse conditions [16]. The anticancer properties of methotrexate are attributed to its ability to induce apoptosis [17,18]. Regrettably, methotrexate-induced apoptosis can also affect healthy liver tissues [10]. ROS signaling can further contribute to methotrexate-induced apoptosis, thereby enhancing its cytotoxic effects [19].

Despite these potential toxicities and adverse effects, methotrexate remains a widely used and preferred first-line antirheumatic drug in many countries due to its affordability and effectiveness in treating various medical conditions. Its inclusion in the “World Health Organization’s List of Essential Medicines” highlights its importance in healthcare systems worldwide. Although concerns exist regarding its impact on the liver and potential tissue damage, the benefits of methotrexate outweigh these risks, making it a valuable treatment option for many patients [4,20,21]. Additionally, scientific reports and meta-analyses have emphasized its superior efficacy compared to other available drugs, further emphasizing its significance in medical treatments [21]. Consequently, efforts are underway to develop strategies that can protect the liver and enhance the overall safety profile of methotrexate in order to address its associated hepatotoxicity [22,23].

The therapeutic properties of medicinal herbs have garnered significant attention in recent years for treating a range of human ailments. These herbs have a broad safety profile and can effectively mitigate the cytotoxic effects of more hazardous drugs. As a result, it has become common practice to combine these compounds with methotrexate-based therapeutic approaches [24].

*Chamaecyparis lawsoniana* (Murr.) Parl., commonly referred to as Lawson’s cypress, is a popular ornamental plant belonging to the Cupressaceae family. It is native to North America and can also be found in several other countries, including Germany, France, the United Kingdom, Australia, and South Africa. This versatile plant has various applications, including in construction and railway sleeper production [25]. It also has a long history of traditional use in treating ailments such as stomach pain, tumors, and lipoma [26]. Previous studies have indicated that extracts from the leaves and bark of this plant have antibacterial, fungicidal, and antioxidant characteristics [27,28]. Nevertheless, until now, no research has been conducted to examine the phytochemical composition of the aerial parts of *C. lawsoniana* or its potential hepatoprotective effects.

Therefore, the main objectives of this study were to determine the chemical profile of the ethanolic extract of *C. lawsoniana* aerial parts (CLAE) and to investigate its potential efficacy in protecting against DILI, specifically an acute methotrexate hepatotoxicity model in rats. Further, its antioxidant, anti-inflammatory, and antiapoptotic properties were also investigated. This was achieved through an in silico approach followed by in vivo validation experiments.

## 2. Materials and Methods

### 2.1. Plant Material and Extraction

The aerial parts of *Chamaecyparis lawsoniana* (A. Murray) Parl. were collected in March 2023 from El-Orman Botanical Garden, located in Giza, Egypt. The taxonomic validation of the plant species was conducted by Eng. Therese Labib, a Plant Taxonomy Consultant at the Ministry of Agriculture and former director of the El-Orman Botanical Garden in Giza, Egypt. At the Herbarium of the Pharmacognosy Department, Faculty of Pharmacy, Zagazig University, a voucher specimen with the code ZU-Ph-Cog-0311 was preserved.

The dried powdered aerial parts (400 g) were macerated with 70% ethanol (3 × 1 L) for extraction. Under reduced pressure, the extract was evaporated to yield 65 g of viscous residue.

### 2.2. Analysis of CLAE Using UPLC-ESI-MS/MS Technique

CLAE (50 mg) was dissolved in a 1 mL solution containing water, methanol, and acetonitrile in a ratio of 50:25:25. The resulting mixture was subjected to vortexing for 2 min, followed by ultrasonication for 10 min. Subsequently, the mixture was centrifuged at 1000 rpm for 10 min. A volume of 50 µL of the sample solution was diluted with reconstitution solvent to a final volume of 1000 µL. From this diluted solution, 10 µL with a concentration of 2.5 µg/µL was prepared for UPLC-ESI-MS/MS analysis in negative mode. The analysis was performed using the ExionLCTM AD UPLC instrument and a TripleTOF 5600+ Time-of-Flight Tandem Mass Spectrometer (AB SCIEX) following the previously described method [29]. As a pre-column, in-line filter disks (0.5 µm × 3.0 mm, Phenomenex^®^, Torrance, CA, USA) were used, while the analytical column was X select HSS T3 (2.5 µm, 2.1 × 150 mm, Waters^®^, 40 °C, Milford, MA, USA). The temperature of the column and the flow rate were set at 40 °C and 0.3 mL/min, respectively. As mobile phases, buffers A and B were used; buffer A is a 5 mM ammonium format buffer, pH 8, containing 1% methanol, and buffer B is composed of 100% acetonitrile. Gradient elution was applied as follows: for 20 min, 90% solvent A and 10% solvent B were used, then for the next 5 min, a mixture of 10% solvent A and 90% solvent B was run, and for the last 3 min, the starting elution mixture was used. The tentative identification of the compounds was carried out based on their retention times (RTs), molecular weight, *m*/*z* of molecular ion [M−H]^−^, and by comparing the accurate mass information from their mass spectrometry (MS) and MS/MS spectra with the MS spectral data generated by the PeakViewTM software version 2.1. The peak area values were estimated using the Extracted Ion Chromatogram Manager in the PeakView software (AB SCIEX, version 1.2.0.3).

### 2.3. Network Pharmacology

#### 2.3.1. Selection of the Bioactive Compounds of CLAE and Associated Targets

The Canonical SMILES formulas of CLAE constituents, identified by LC-MS, were collected from the PubChem database (https://pubchem.ncbi.nlm.nih.gov/, accessed on 3 July 2023) or using ChemDraw v22.0.0.22 (PerkinElmer Informatics, Inc., Buckinghamshire, UK) and were then submitted to the SwissADME web tool (http://www.swissadme.ch/, accessed on 7 July 2023) [30] to retrieve their pharmacokinetic parameters. The selection of compounds was based on the Lipinski’s rule of five and a bioavailability score of ≥0.55.

The molecular targets associated with the bioactive constituents of CLAE were explored using the PharmMapper (https://www.lilab-ecust.cn/pharmmapper/, accessed on 11 July 2023) [31] and SwissTargetPrediction databases (http://www.swisstargetprediction.ch/, accessed on 11 July 2023) [32] and then authenticated in the UniProt database (https://www.uniprot.org/, accessed on 11 July 2023) [33]. The protein names were standardized, and the duplicate targets were eliminated.

#### 2.3.2. Identification of DILI-Associated Targets

GeneCards (https://www.genecards.org/, accessed on 17 July 2023) [34,35], DisGeNeT (https://www.disgenet.org/search, accessed on 17 July 2023) [36], and Online Mendelian Inheritance in Man (OMIM, https://www.omim.org/, accessed on 17 July 2023) [37] were used for the collection of the DILI-related targets using “Drug-induced hepatotoxicity” as the keyword, then the UniProt IDs and gene symbols of the collected targets were obtained from UniProt and the duplicate targets were removed.

#### 2.3.3. The Establishment of the Protein–Protein Interaction (PPI) and Compound–Target Networks

In Microsoft Excel, the overlaps between the bioactive CLAE components and DILI targets were determined and then illustrated as a Venn diagram. The STRING database v12.0 (https://string-db.org/, accessed on 27 July 2023) [38] was used to construct a PPI network of the overlapped targets at a confidence level of >0.7. Following the construction of the PPI network, a compound–target network was also established connecting the bioactive compounds of CLAE with the overlapping targets. The Cytoscape 3.9.1 software program (NIGMS, Bethesda, MD, USA) [39] was employed to display the networks. The targets and compounds were ranked based on the Degree value using the CytoHubba plugin in Cytoscape [40].

#### 2.3.4. Analysis of Gene Ontology and KEGGs Pathway Enrichment

The Database for Annotation, Visualization, and Integrated Discovery (DAVID) (https://david.ncifcrf.gov/tools.jsp, accessed on 28 July 2023) [41] was employed to conduct the Gene Ontology (GO) analysis and Kyoto Encyclopedia of Genes and Genomes (KEGGs) pathway enrichment. A significance level of *p* < 0.05 was employed as a cutoff. Homo sapiens (Human) was selected as the organism, and the data sources GO biological process, GO cellular component, GO molecular function, and KEGGs were chosen. The findings were presented in the form of horizontal bar plots using the SRPlot online toolkit (http://www.bioinformatics.com.cn/en, accessed on 28 July 2023).

### 2.4. Molecular Docking

To further validate the results obtained from the network analysis, molecular docking analysis was performed to evaluate the potential binding activity and interaction between the three highly ranked compounds, namely sequoiaflavone, 3-hydroxysandaracopimaric acid, and 3,7-dimethylquercetin, and the top eight core targets.

#### 2.4.1. Protein and Ligand Preparation

The three-dimensional (3D) crystal structures of the proteins, including cellular tumor antigen p53 (TP53; PDB ID: 8DC4/2.40 Å) [42], interleukin-6 (IL6; PDB ID: 4NI9/2.55 Å) [43], tumor necrosis factor (TNF-α; PDB ID: 2AZ5/2.10 Å) [44], heat shock protein 90-alpha (HSP90AA1; PDB ID: 8AGI/2.10 Å) [45], epidermal growth factor receptor (EGFR; PDB ID: 7T4I/2.61 Å) [46], interleukin-1 beta (IL1B; PDB ID: 1T4Q/2.10 Å) [47], apoptosis regulator Bcl-2 (BCL2; PDB ID: 7LHB/2.07 Å) [48], and caspase-3 (CASP3; PDB ID: 3KJF/2.00 Å) [49], were attained from the Protein Data Bank (http://www.rcsb.org, accessed on 29 July 2023) [50]. The Biovia Discovery Studio visualizer v21.1.0.20298 [51] was employed to eliminate the co-crystallized ligands, water molecules, ions, and repeated chains. Then, the Dock Prep module in the USCF Chimera 1.17.3 software [52] was used to modify the protein structures by adding polar hydrogens and Gasteiger charges. The modified structures were saved as PDBQT protein receptor files.

The 3D structures of the selected bioactive compounds of CLAE were retrieved from the PubChem database and subsequently converted to dockable pdbqt formats using OpenBabel 2.4.1 [53].

#### 2.4.2. Determination of the Grid Coordinates of the Active Sites

For each protein, a grid box was placed on the active site to determine the corresponding grid coordinates using the Auto Dock Vina suite in the USCF Chimera software v.1.17.3. However, for proteins IL6 and IL1B, no co-crystallized ligands were available. As a result, the Computed Atlas for Surface Topography of Proteins server (CASTp; http://sts.bioe.uic.edu/castp/index.html, accessed on 29 July 2023) [54] was used first to predict the active pocket, followed by the determination of the respective coordinates. The centers and sizes of the grid boxes, as well as the amino acid residues of the active sites, are revealed in Table S1.

#### 2.4.3. Docking Simulation and Visualization

The molecular docking of the key components onto target proteins was processed using AutoDock Vina 1.1.2. The default docking parameters were set with an energy range of 4 and an exhaustiveness of 8 in order to generate 10 distinct poses of ligand molecules. The docking scores were expressed in kcal/mol, with a lower score indicating a stronger binding affinity. For each ligand, the docked pose with the best score and least root mean square deviation (RMSD) value was selected. Additionally, for the confirmation process of the active site, the co-crystallized ligands for TNF, HSP90AA1, EGFR, Bcl-2, and CASP3 were also re-docked. The visualization of the molecular interactions between proteins and ligands was achieved using Maestro v13.6.122 software (Schrödinger Release 2023-3: Maestro, Schrödinger, LLC, New York, NY, USA, 2023) and the Biovia Discovery Studio Visualizer v21.1.0.20298 (BIOVIA Dassault Systemes, San Diego, CA, USA).

### 2.5. In Vivo Experiments

#### 2.5.1. Animals

Twenty-four adult male Wistar rats, weighing 210 ± 20 g, were purchased from the animal unit in the Faculty of Veterinary Medicine, Zagazig University, Zagazig, Egypt. Throughout the adaptation period and the experiment, the rats were housed in the animal house unit in the Faculty of Pharmacy, Zagazig University, Zagazig, Egypt, and maintained under optimal conditions of temperature (22 ± 3 °C), humidity (60 ± 10%), and a 12/12 h light/dark cycle. Water and a normal chow diet were accessible ad libitum.

#### 2.5.2. Ethical Statement

The followed research protocol here was approved by the Institutional Animal Care and Use Committee at Zagazig University, Egypt, and given the approval number ZU-IACUC/3/F/207/2023. The recommendations of the Weather All report and the National Institutes of Health Guide for the care and use of laboratory animals were strictly followed.

#### 2.5.3. Drugs and Vehicles

MTX was obtained from MYLAN (Haupt Pharma GmbH, Münster, Germany), and tween 80 was purchased from Sigma–Aldrich (St Louis, MO, USA). CLAE was prepared in commercially available corn oil with 10% tween 80. All other used chemicals are of analytical grade.

#### 2.5.4. Experimental Protocol

##### Induction of MTX-Hepatotoxicity

Following two weeks of acclimatization, the experiment was launched. Hepatotoxicity was developed in all groups (except for the control one) by a single i.p injection of 20 mg/kg MTX [11] on the fifth day of the experiment. For the control, the rats received a single i.p injection of saline as an MTX vehicle.

##### Study Groups

The animals were randomly assigned into four groups (n = 6 rats each) as follows; the control group (animals received a single i.p injection of saline on the fifth day of the experiment plus 10% tween 80 in corn oil, as the extract vehicle, by gavage throughout the experiment), the MTX vehicle group (animals received a single i.p injection of MTX on the fifth day of the experiment plus 10% tween 80 in corn oil by gavage throughout the experiment), and the CLAE 200 and CLAE 400 groups (animals received a single i.p injection of MTX on the fifth day of the experiment plus CLAE in 10% tween 80/corn oil throughout the experiment at 200 and 400 mg/kg/day, gavage, respectively). CLAE or vehicle administration began from the start of the experiment and continued for five days after the MTX injection (for a total experiment period of 10 days).

#### 2.5.5. Blood and Tissue Samples Preparation

At the closure of the experiment, blood samples were withdrawn from retro-orbital plexus by means of heparinized microcapillary tubes and under light anesthesia with sodium pentobarbital (50 mg/kg, i.p) [55]. The collected blood samples were allowed to stand and clot for 30 min at 4 °C and were then centrifuged at 3000× *g* at 4 °C for another 20 min. Serum was aspirated, aliquoted, and immediately stored at −80 °C for later biochemical analysis. Euthanasia was ensured by cervical dislocation, liver was then excised immediately, rinsed with ice cold saline, and blotted dry on tissue paper. Each collected liver was divided into two portions: one of them was fixed 10% formalin for histopathological examination, while the other was flash-frozen using liquid nitrogen and then stored at −80 °C for later assays.

#### 2.5.6. Assessment of Serum Biomarkers

##### Liver Function Biomarkers

To assess liver function, alanine transaminase (ALT), aspartate transaminase (AST), and alkaline phosphatase (ALP) were measured in serum using commercially available colorimetric kits from Spinreact Co. (Girona, Spain). The manufacturer’s instructions were followed precisely, and measurements were carried out in duplicate.

#### 2.5.7. Assessment of Hepatic Biomarkers

##### Oxidative Stress Biomarkers

The hepatic malondialdehyde (MDA) level, as an index of lipid peroxidation, as well as the hepatic reduced glutathione (GSH) level and superoxide dismutase (SOD) activity, as indicators of the hepatic antioxidant capacity, were measured in liver homogenates using Bio-Diagnostic Co. (Giza, Egypt) colorimetric kits. The measurements were performed in duplicates and in accordance with the manufacturer’s instructions.

##### Proinflammatory Cytokines

Proinflammatory cytokine, TNF-*α*, was measured in liver homogenates using a rat TNF-α ELISA kit purchased from BT LAB (Shanghai, China). The measurements were conducted in duplicates, following the instructions provided by the manufacturer.

##### Apoptotic Biomarkers

For the hepatic apoptosis assessment, apoptotic regulators Bcl-2 and Bax, as well as the proapoptotic caspase-3 content, were measured in liver homogenates using rat ELISA kits (BCL2L1, BAX, and CASP3, respectively) BT LAB (Shanghai, China). All assays were conducted in duplicate as per the manufacturers’ instructions.

#### 2.5.8. Immunohistochemical Staining

Serial sections of 4 μm thicknesses were cut from paraffin blocks of livers and then further processed for immunohistochemical staining as follows: (1) Sections were immersed into a 10 mM citrate buffer (pH 6.0) and heated at 98 °C in a water bath for 30 min and then washed with water, (2) 3% hydrogen peroxide in methanol was added to sections for 15 min to block the endogenous peroxidase activity, (3) Sections were incubated with horse serum for 10 min at room temperature to block non-specific binding, (4) Sections were incubated overnight at 4 °C with anti-p53 polyclonal antibody (Invitrogen, Carlsbad, CA, USA) at 1:100 dilution as a proapoptotic biomarker, or with anti-Bcl-2 (Santa Cruz Biotechnology Inc., Paso Robles, CA, USA) at 1:50 dilution as an antiapoptotic biomarker, (5) Sections were incubated with secondary biotinylated antibody and avidin–biotin complex (Vectastain® ABC-peroxidase kit, Vector Laboratories, Burlingame, CA, USA, (6) The color was developed by adding 3,3-diaminobenzidine (DAB) solution, and, (7) Finally, the images were captured using light microscopy (LEICA ICC50W) in the Anatomy and Embryology department by an expert pathologist who screened the entire section and captured the most representative images for each group. The images were analyzed using the Image J software plugin (version 1.53v), immunohistochemistry (IHC) profiler, to calculate the percentage of positive areas (areas stained with brown color) according to the method previously described [56].

#### 2.5.9. Histopathological Examination

Paraffinized livers were sectioned at 5 μm thickness using a microtome (Leica RM 2155, Newcastle upon Tyne, UK). Then, sections were deparaffinized in xylene, gradually hydrated, and then stained with hematoxylin and eosin (H&E). An expert pathologist, blinded to the study groups, screened the entire section and captured the most representative images for each group using light microscopy (LEICA ICC50W) in the Anatomy and Embryology department. Portal tract inflammation was graded as none, mild, moderate, and severe (0–3), where 0 = no portal inflammation, 1 = sprinkling of inflammatory cells in 1/3 of portal tracts, 2 = increased inflammatory cells in 1/3–2/3 of portal tracts, and 3 = dense packing of inflammatory cells in 0.2/3 of portal tracts [57].

#### 2.5.10. Statistical Analysis

All data were represented as mean ± standard error of the mean (SEM). Statistical analysis was conducted using Graph pad prism software version 9.4.1 (681) (Graph Pad Software Inc., La Jolla, CA, USA). The statistical significance of differences between the groups was performed using a one-way analysis of variance (ANOVA) followed by Tukey’s Post hoc test. A significant difference was assumed for values of *p* less than 0.05. For histology scoring, the statistical significance of differences between groups was performed using the Kruskal–Wallis test followed by Dunn’s multiple comparisons test.

## 3. Results

**Figure 1:**
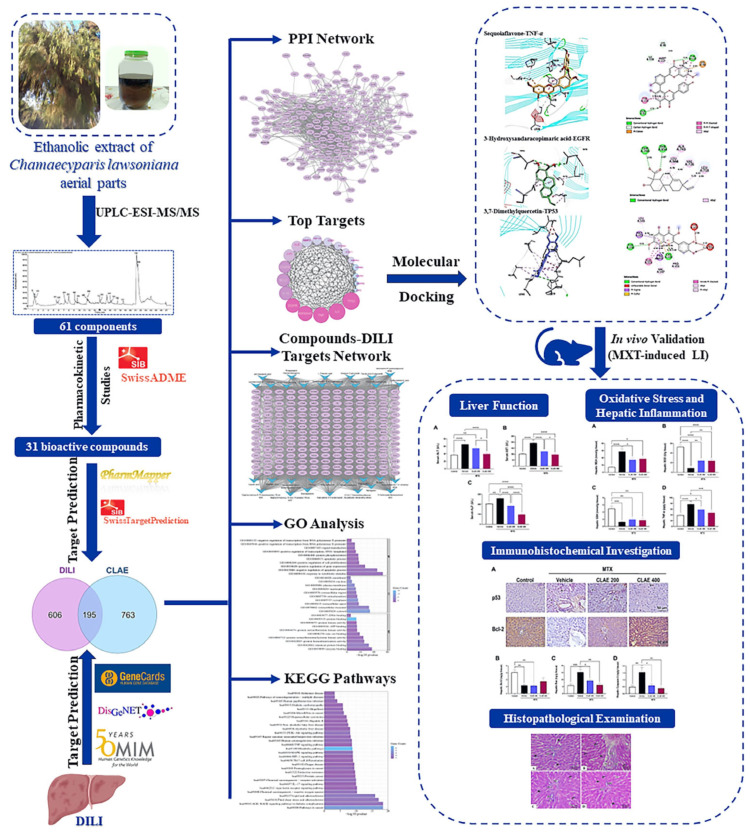
A flowchart depicting the experimental design of this study, encompassing phytochemical, network pharmacological, molecular docking, and in vivo experimental studies to explore the impact of CLAE in DILI.

The present investigation implemented a systematic experimental approach (Figure 1) to reveal the chemical composition of CLAE, utilizing ultra-performance liquid chromatography–electrospray tandem mass spectrometry (UPLC-ESI-MS/MS). The identified compounds were further analyzed through in silico techniques, including network pharmacology and molecular docking analysis, to investigate their interactions with the DILI molecular targets. To validate the findings in vivo, a rat model of liver injury induced by MTX was employed, followed by subsequent functional and immunohistochemical assessments.

### 3.1. UPLC-ESI-MS/MS Profiling

According to MS mass, MS^2^ fragmentation data and patterns, and literature reports, 61 chemical constituents were identified, categorized into flavonoids and glycosides, phenolic, diterpene, carboxylic, sugar acids, fatty acids, lignans, and other compounds. Retention time, pseuomolecular ion peak [M-H]^−^, MS^2^, and the related literature of the identified metabolites of CLAE are listed in Table 1. Figure S1 shows the total ion chromatogram (TIC) of CLAE in negative mode.

**Table 1:** Phytochemical profiling of the ethanolic extract of *Chamaecyparis lawsoniana* aerial parts by LC-ESI-MS/MS in negative mode.

No.	Rt.	[M-H]^−^	MS^2^ Fragments (*m*/*z*)	Tentative Identification	Class	Ref.
1.	1.068	133.014	115, 71	Malic acid	Carboxylic acid	[58]
2.	1.119	173.045	155, 111, 137, 73, 93	Shikimic acid	Carboxylic acid	[59]
3.	1.158	135.030	117, 99, 73, 75	L-Threonic acid	Sugar acid	[59]
4.	1.163	329.091	167	Vanillic acid glucoside	Phenolic acid glycoside	[60]
5.	1.183	191.056	173, 85	Quinic acid	Carboxylic acid	[29]
6.	1.211	335.054	299, 191, 137	Caffeoylshikimic acid	Phenolic acid derivatives	[61]
7.	1.237	377.086	341	Disaccharid adduct	Disaccharid	[62]
8.	1.275	315.071	153	Protocatechuic acid hexoside	Phenolic acid glycoside	[29]
9.	1.301	355.116	193, 149, 175, 134	Ferulic acid-*O*-glucoside	Phenolic acid glycoside	[63]
10.	1.379	341.109	59, 71, 89, 101, 113, 143	Sucrose	Disaccharid	[62]
11.	1.405	337.092	191, 163, 119	Coumaroylquinic acid	Phenolic acid derivatives	[64]
12.	1.458	357.119	195	Dihydro-ferulic acid hexoside	Phenolic acid glycoside	[65]
13.	4.162	507.164	345	Syringetin-3-*O*-glucoside	Flavonol glycoside	[66]
14.	5.339	489.143	313, 283	5,7-Dihydroxy-8,2’-dimethoxyflavone 7-glucuronide	Flavone glucuronide	[67]
15.	5.537	385.186	223, 153	Roseoside	Norisoprenoid glucoside	[64]
16.	5.564	385.186	223, 179	Sinapoyl D-glucoside	Phenolic acid glycoside	[68]
17.	5.645	431.192	385, 223, 153	Roseoside (formate adduct)	Norisoprenoid glucoside	[64]
18.	5.648	593.153	447, 431, 285	kaempferol-3-*O*-glucoside-7-*O*-rhamnoside	Flavonol glycoside	[69]
19.	5.751	623.158	487, 477, 461, 443, 315, 297	Verbascoside	Phenylethanoid glycosides	[70]
20.	5.775	525.197	329, 507	Tricin-4′-O-(erythro-β-guaiacylglyceryl) ether (Salcolin A)	Flavone derv.	[71]
21.	5.777	623.160	477, 315	Isorhamnetin-3-*O*-rutinoside	Flavonol glycoside	[72]
22.	5.777	623.160	461, 477	Isorhamnetin 3-*O*-glucoside-7-*O*-rhamnoside	Flavonol glycoside	[73]
23.	6.110	373.149	327	Pinopalustrin (Nortrachelogenin)	Dibenzylbutyrolactone lignan	[74]
24.	6.433	609.146	463, 447, 301	Quercetin 3-rhamnoglucoside	Flavonol glycoside	[75]
25.	6.615	463.088	301, 300, 179, 271, 255, 151	Quercetin-3-*O*-glucoside	Flavonol glycoside	[64]
26.	6.633	609.111	447, 285	kaempferol dihexoside	Flavonol glycoside	[76]
27.	6.860	593.152	431, 385, 311, 269	Apigenin diglucoside	Flavone glycoside	[77]
28.	6.882	363.144	315, 179, 167	(7R,8R)-3-Methoxy-3’,4,7,9,9’-pentahydroxy-8,4’-oxyneolignan	Lignan	[78]
29.	7.264	447.092	301, 179, 151, 271	Quercitrin (Quercetin -3-*O*-rhamnoside)	Flavonol glycoside	[64]
30.	7.316	477.103	315, 314, 285	Isorhamnetin 3-*O*-Glucoside	Flavonol glycoside	[79]
31.	7.416	327.217	327, 229, 211, 171, 113	9,12,13-trihydroxyoctadeca-10,15-dienoic acid (Malyngic acid)	Fatty Acid	[80]
32.	7.518	287.056	259, 151	Dihydrokaempferol (Aromadendrin)	Flavanonol	[72]
**33.**	7.538	699.135		Agathisflavone -*O*-hexoside	Biflavonoid glycoside	[81]
34.	7.586	577.156	269, 225, 201, 149	Apigenin 7-*O*-neohesperidoside (rhoifolin)	Flavone glycoside	[82]
35.	7.861	329.138	314, 299	3,7-dimethylquercetin	Flavonol	[83]
36.	7.862	341.141	311, 283, 257	4’,5,6,7-Tetramethoxyflavone (Scutellarein tetramethyl ether)	Flavone	[84]
37.	7.887	435.149	273, 167	Phlorizin (phloretin glucoside)	Dihydrochalcone glycoside	[29]
38.	7.976	461.107	461, 299, 284	Dihydro-methoxyisoflavone *O*-hexoside (Tectoridin)	Flavone glycoside	[85]
39.	8.052	461.108	315, 314	Isorhamnetin-*O*-rhamnoside	Flavonol glycoside	[86]
40.	8.220	519.187	459, 357, 315, 314, 299, 285	Hexosyl-acyl-isorhamnetin	Flavonol glycoside	[87]
41.	8.283	417.082	285, 284, 255	Kaempferol-3-*O*-arabinoside	Flavonol glycoside	[88]
42.	8.692	557.244	539, 509, 361	Secoisolariciresinol guaiacylglyceryl ether	Butanediol lignan	[89]
43.	8.865	555.224	525, 507, 329, 195, 165	Lariciresinol-4’-guaiacylglyceryl ether	Tetrahydrofuranolignan	[89]
44.	9.366	537.273	417, 375, 399	Agathisflavone	Biflavonoid	[81]
45.	9.639	543.276	335	Pharboside C	Diterpene acid glycoside	[90]
46.	9.948	271.062	151	Naringenin	Flavanone	[72]
47.	10.454	137.024	93	Protocatechualdehyde	Phenolic aldehyde	[91]
48.	10.955	521.087	329, 359	Lariciresinol glucoside	Tetrahydrofuranolignan glycoside	[92]
49.	11.580	551.096	457, 431, 413, 389, 345	7-*O*-methylamentoflavone (Sequoiaflavone)	Biflavonoid	[93]
50.	11.629	551.097	457, 431, 413, 389, 390, 345	4′-*O*-methylamentoflavone (Bilobetin)	Biflavonoid	[94]
51.	14.081	333.258	315	8alpha-8-Hydroxy-12-oxo-13-abieten-18-oic acid	Diterpene acid	[95]
52.	14.433	302.911	259, 219	Copalic acid	Diterpene acid	[74]
53.	16.038	565.115	533, 389, 374	Isoginkgetin (4′,4″ dimethylamentoflavone)	Biflavonoid	[94]
54.	16.416	564.773	471, 445, 403	Robustaflavone 7,4′-dimethyl ether	Biflavonoid	[94]
55.	16.715	357.099	342, 313	Matairesinol	Dibenzylbutyrolactone lignans	[96]
56.	17.152	359.222	344, 313	Cyclolariciresinol	Aryltetralin diol lignan	[89]
57.	18.682	329.175	285, 313, 311	Carnosol	Phenolic diterpene	[74]
58.	21.153	317.212	299, 205	3-Hydroxysandaracopimaric acid	Diterpene acid	[97]
59.	21.191	317.212	299	12alpha-hydroxy-8,15-isopimaradien-18-oic acid	Diterpene acid	[98]
60.	21.202	301.218	253, 205	ent-kaurenoic acid	Diterpene acid	[99]
61.	21.269	715.328	641, 375, 301	Ganoleucoin J	lanostane triterpenoid	[100]

#### 3.1.1. Identification of Phenolic, Carboxylic, Sugar, Diterpene Acid and Fatty Acids

According to the UPLC-ESI-MS/MS analysis conducted in negative mode, CLAE displayed a diverse range of acids that were classified into various categories, including phenolic acid conjugates, carboxylic acids, sugar acids, diterpene acids, and fatty acids.

Phenolic acid conjugates were predominantly observed as phenolic acid hexosides, such as compounds **4**, **8**, **9**, and **12**, which released hexosyl (162 Da) to produce corresponding phenolic acids, including vanillic, protocatechuic, ferulic, and dihydroferulic acids. Other phenolic acid conjugates, such as caffeoylshikimic acid **6** and coumaroylquinic acid **11**, were also identified.

In addition to these, carboxylic acids, such as malic and shikimic acids, sugar acid as L-threonic acid, diterpene acids, including 8alpha-8-Hydroxy-12-oxo-13-abieten-18-oic acid, copalic acid, 3-hydroxysandaracopimaric acid, 12alpha-hydroxy-8,15-isopimaradien-18-oic acid, and ent-kaurenoic acid, and diterpene acid glycoside pharboside C, as well as fatty acids, such as 9,12,13-trihydroxyoctadeca-10,15-dienoic acid, were also characterized. Generally, the primary fragmentation pathway for these acids involved the loss of CO (28 Da), CO_2_ (44 Da), and H_2_O from the deprotonated peak [M-H]^−^.

#### 3.1.2. Identification of Flavonoid and Glycosides

Flavonoid aglycones and glycosides are considered the major compounds detected in CLAE; these compounds belong to different subclasses such as flavonol, flavone, flavanonol, biflavonoid, dihydrochalcone, and flavanone.

Biflavonoids represent the majority of the subclasses in the extract, where six biflavonoids were tentatively identified, including three 3′, 8″ biapigenin-type biflavones (IC3′–IIC8″) as 7-*O*-methylamentoflavone **49**, 4′-*O*-methylamentoflavone **50**, and Isoginkgetin **53**, one 3′, 6″ biapigenin-type biflavone (IC3′–IIC6″) as robustaflavone 7,4′-dimethyl ether **54**, and two 6, 8″ biapigenin-type biflavones (IC6–IIC8″) as agathisflavone-*O*-hexoside **33** and agathisflavone **44**. Compounds **49**, **50**, and **53** are amentoflavone-type biflavones, and they underwent a similar fragmentation pathway. The [M-H]^−^ ion of compound **49** at *m*/*z* 551 produced several characteristic daughter ions, such as the [M-H-C_6_H_6_O]^−^ ion at *m*/*z* 457, which is coming from the neutral loss of phenol on flavonoid part II, [M-H-C_7_H_4_O_2_]^−^ ion at *m*/*z* 431, which was attributed to the ^0,2^IIA-ion, [M-H-C_7_H_6_O_3_]^−^ ion at *m*/*z* 413 which corresponded to the ^0,2^IIA^−^-H_2_O ion, [M-H-C_9_H_6_O_3_]^−^ ion at *m*/*z* 389 ion which corresponded to the base peak, which illustrated that the product ion passed a retro cyclization fragmentation, including the 0 and 4 bonds on flavonoid part II, and [M-H-C_10_H_6_O_5_]^−^ ion at *m*/*z* 345 which corresponded to the ^0,4^IIA^−^-CO_2_ ion. Compounds **50** and **53** also yielded diagnostic fragments for this type of biflavone. Basically, the most important diagnostic fragmentation -ve ESI mode of amentoflavone-type biflavones is that involving the cleavage of the C–ring of flavonoid part II at position 0/4. The MS^2^ fragmentation pathways of IC3′–IIC6″ linked biflavones, such as robustaflavone 7,4′-dimethyl ether **54**, displayed similarities and differences in comparison with amentoflavone-type biflavones. Compound **54** produced fragments at *m*/*z* 471, 445, and 403 in a similar way as amentoflavone-type biflavones. But the chances are greater in the case of robustaflavone type for the cleavage of C–ring to occur on flavonoid part I, such as at position 1/4 and 1/3, and after retro cyclization, which produced the 1,4IB-ion at *m*/*z* 427, 1,3IB^-^ ion at *m*/*z* 401.

Other flavonoid aglycones were tentatively identified as flavanonol (dihydrokaempferol **32**), flavonol (3,7-dimethylquercetin **35**), flavone (scutellarein tetramethyl ether **36**), and flavanone (naringenin **46**). The identification of these aglycones was established by the corresponding [M-H]^−^ as well as the MS^2^ fragmentation pattern for each compound.

Flavonoids are mostly present in the form of glycosides, which are easily cleaved in MS^2^ fragmentation, producing the corresponding aglycone. Three peaks related to Kaempferol were detected at [M-H]^−^ at *m*/*z* 593, 609, and 417, they gave a fragment at *m*/*z* 285, corresponding to the aglycone Kaempferol, which attributed to the elimination of glucose and rhamnose (compound **18**), two molecules of glucose (compound **26**), and arabinose (compound **41**). Peaks **21**, **22**, **30**, **39**, and **40** exhibited the same base peak at *m*/*z* 315 corresponding to the isorhamnetin aglycone through the neutral loss of rutinosyl (308 Da), indicating the presence of isorhamnetin-3-*O*-rutinoside 23, glucosyl, and rhamnosyl (162, 146 Da), indicating the presence of isorhamnetin 3-*O*-glucoside-7-*O*-rhamnoside 24, glucosyl (162 Da), confirming isorhamnetin 3-*O*-glucoside, the loss of rhamnosyl (146 Da) in the case of isorhamnetin-*O*-rhamnoside **41**, and the loss of acylhexosyl (204 Da) in hexosyl-acyl-isorhamnetin **40**. In a similar way, quercetin glycosides (compounds **24**, **25**, and **29**), apigenin glycosides (compounds **27** and **34**), syringetin-3-*O*-glucoside **13**, 5,7-Dihydroxy-8,2’-dimethoxyflavone 7-glucuronide **14**, phloretin glucoside **37**, and diosmetin 7-*O*-glucoside were tentatively identified.

Other flavonoid conjugates were detected as compound **20** of the molecular ion peak [M-H]^−^ at *m*/*z* 525, and MS^2^ fragmentation produced a characteristic peak for the aglycone tricin and identified as salcolin A (tricin-4′-*O*-(erythro-*β*-guaiacylglyceryl) ether).

#### 3.1.3. Identification of Lignans and Their Glycosides

Different classes of lignans and glycosides were identified in the extract as dibenzylbutyrolactones (**23**, **55**), butanediol (**42**), tetrahydrofurano (**43**, **48**), aryltetralin diol lignans (**56**), and neolignan (**28**); they exhibited different fragmentation patterns which were compared with the reported data.

#### 3.1.4. Identification of Miscellaneous Compounds

Disaccharide (sucrose), norisoprenoid glucoside (roseoside), phenylethanoid glycosides (verbascoside), phenolic aldehyde (protocatechualdehyde), phenolic diterpene (carnosol), and lanostane triterpenoid (ganoleucoin J) were also identified.

### 3.2. Network Pharmacology-Based Analysis

#### 3.2.1. Identification of Bioactive Constituents of CLAE

In order to identify the potential bioactive components, a total of 54 secondary metabolites of CLAE were subjected to screening for their pharmacokinetic and drug-likeness properties, as detailed in Table S2. Among these compounds, 31 exhibited high bioavailability scores (OB ≥ 0.55) and satisfied Lipinski’s rule of five, a widely accepted criterion for assessing drug likeness. Consequently, these 31 compounds were selected for further investigation, outlined in Table S3.

#### 3.2.2. Determination of the Overlapping Molecular Targets of CLAE Bioactive Compounds and DILI

**Figure 2:**
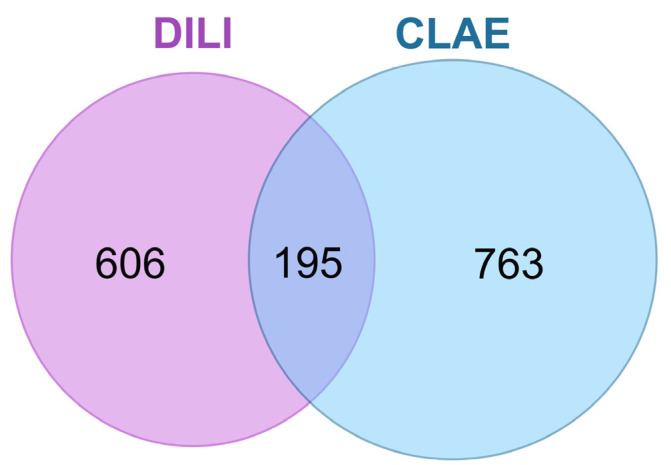
Overlapping molecular targets between DILI and CLAE bioactive compound. CLAE, *Chamaecyparis lawsoniana* aerial parts extract; DILI, drug-induced liver injury.

In order to ascertain the molecular targets related to the bioactive components of CLAE, the databases PharmMapper and SwissTargetPrediction were employed. Following the elimination of duplicates, a total of 958 targets were yielded (Table S4). Subsequently, the DILI-associated molecular targets were identified from three disease-related databases: DisGeNeT, GeneCards, and OMIM. After removing duplicates, 801 targets were obtained from an initial 1114 (Table S5). Of these targets, 195 (Table S6) were found to overlap with the 958 targets associated with CLAE bioactive compounds (Figure 2).

#### 3.2.3. PPI Network of the Common Targets

**Figure 3:**
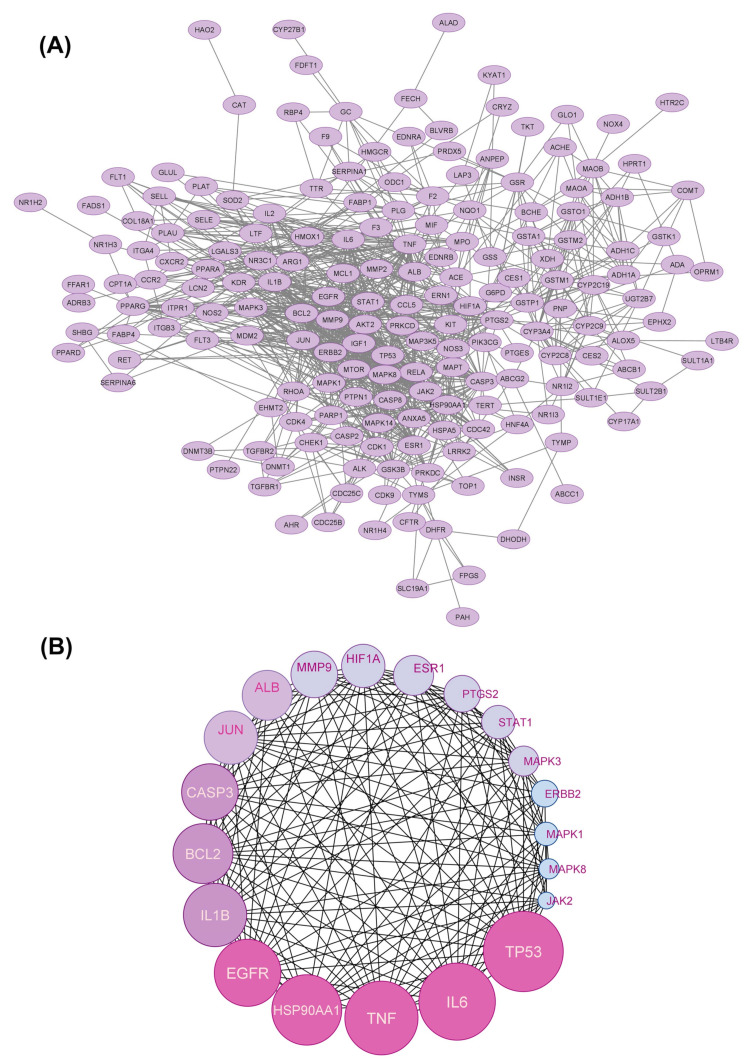
Protein–protein interaction (PPI) network of CLAE molecular targets associated with DILI. (**A**) PPI network. (**B**) Top 20 targets in the PPI network ranked by their Degree values.

To comprehend the hepatoprotective mechanism of CLAE against DILI, the interactions between the common target proteins were analyzed. The 195 overlapping targets were submitted into the STRING database to generate an interconnected network that shows the correlations among these targets. After removing the disconnected nodes, the entire network displayed a total of 185 targets (Figure 3A).

A Degree value-based ranking was performed on the core targets in the PPI network, which was determined by the number of connecting edges. The complete ranking of all the genes can be found in Table S7, whereas the top 20 targets are presented in Figure 3B and Table 2. TP53, IL6, TNF-*α*, HSP90AA1, EGFR, IL1B, BCL2, and CASP3 are among the top eight targets.

#### 3.2.4. Top CLAE Compounds Associated with DILI Targets

In Cytoscape, a compound–target network (Figure S2) was constructed to find out the most significant CLAE compounds related to the 195 DILI targets. These compounds were subsequently arranged by their Degree value (Table 3). The top three compounds were sequoiaflavone, 3-hydroxysandaracopimaric acid, and 3,7-dimethylquercetin.

#### 3.2.5. Enrichment Analysis of the Common Targets

**Figure 4:**
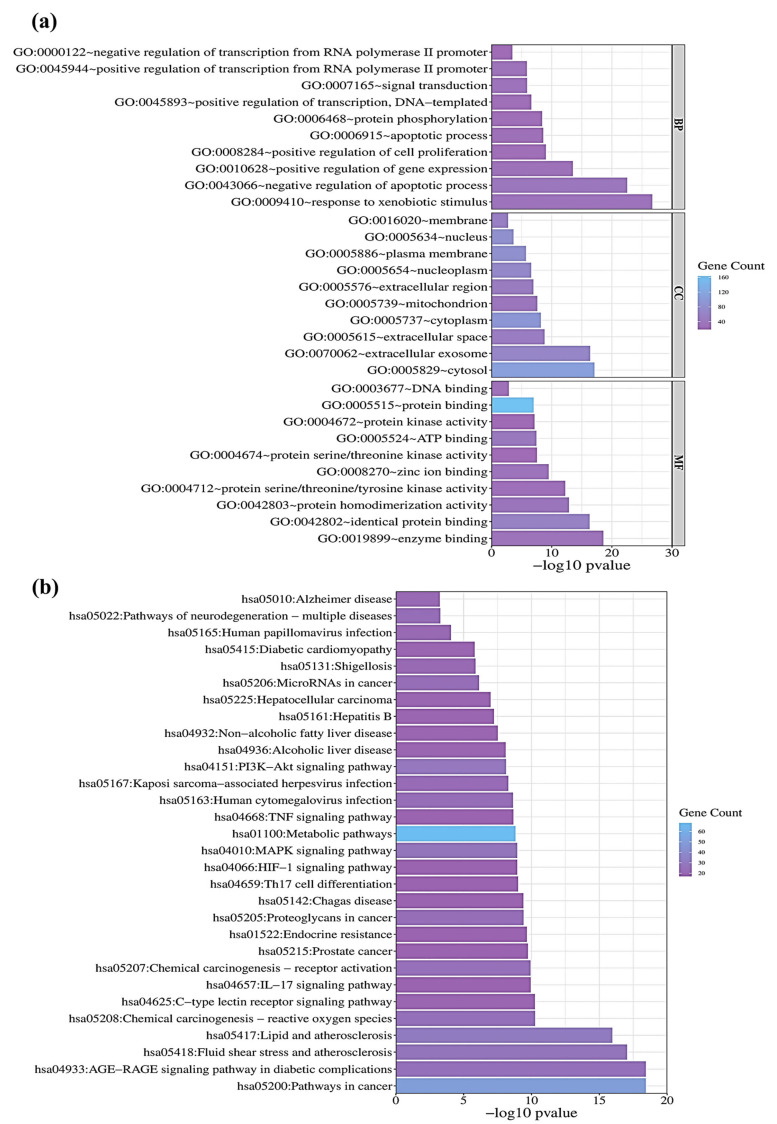
Enrichment analysis compound–disease interacting proteins, (**a**) GO analysis, (**b**) KEGGs pathways. The Degree of color intensity is directly proportional to the number of genes, with intense violet representing the highest level.

The present study conducted an enrichment analysis to confirm the relevant characteristics of the 195 disease–compound common targets on biological and functional levels. The GO analysis yielded a total of 722 GO items, comprising biological processes (BPs), cellular components (CCs), and molecular functions (MFs) with *p* < 0.05. Bar graphs were generated for the top 10 GO items, as illustrated in Figure 4a. The most prominent BP involved the response to xenobiotic stimulus, negative regulation of the apoptotic process, and the xenobiotic metabolic process. The top CC categories were cytosol, extracellular exosome, and macromolecular complex, while the top MF categories comprised enzyme binding, identical protein binding, and protein homodimerization activity. Supplementary Tables S8–S10 provide detailed information on the GO analyses.

Additionally, KEGGs pathway enrichment analysis (*p* < 0.05) was performed on the 195 common targets of CLAE and DILI to identify the potential hepatoprotective pathways. The top 30 pathways, including pathways in cancer, the AGE-RAGE signaling pathway in diabetic complications, fluid shear stress, and atherosclerosis, are shown in Figure 4b based on the number of enriched genes, fold changes, and *p* value. The results of the KEGGs pathway are represented in detail in Table S11.

### 3.3. Molecular Docking Simulation

In order to assess the binding affinity of CLAE compounds to the key target proteins associated with DILI pathogenesis, a molecular docking analysis was conducted using AutoDock Vina software v.1.1.2. The analysis focused on the top three CLAE compounds: sequoiaflavone, 3-hydroxysandaracopimaric acid, and 3,7-dimethylquercetin (Table 3), and the top eight DILI targets: TP53, IL6, TNF-*α*, HSP90AA1, EGFR, IL1B, BCL2, and CASP3 (Table 2). The ligand molecules were docked within the designated grid box that was generated around the active site of each protein.

**Figure 5:**
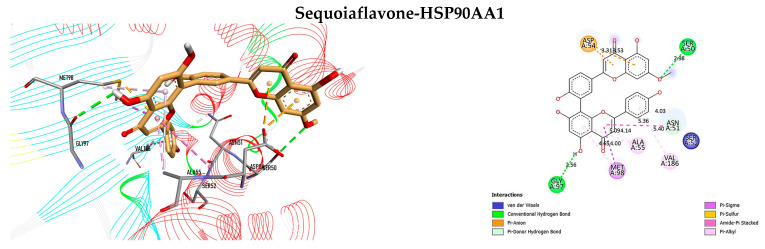

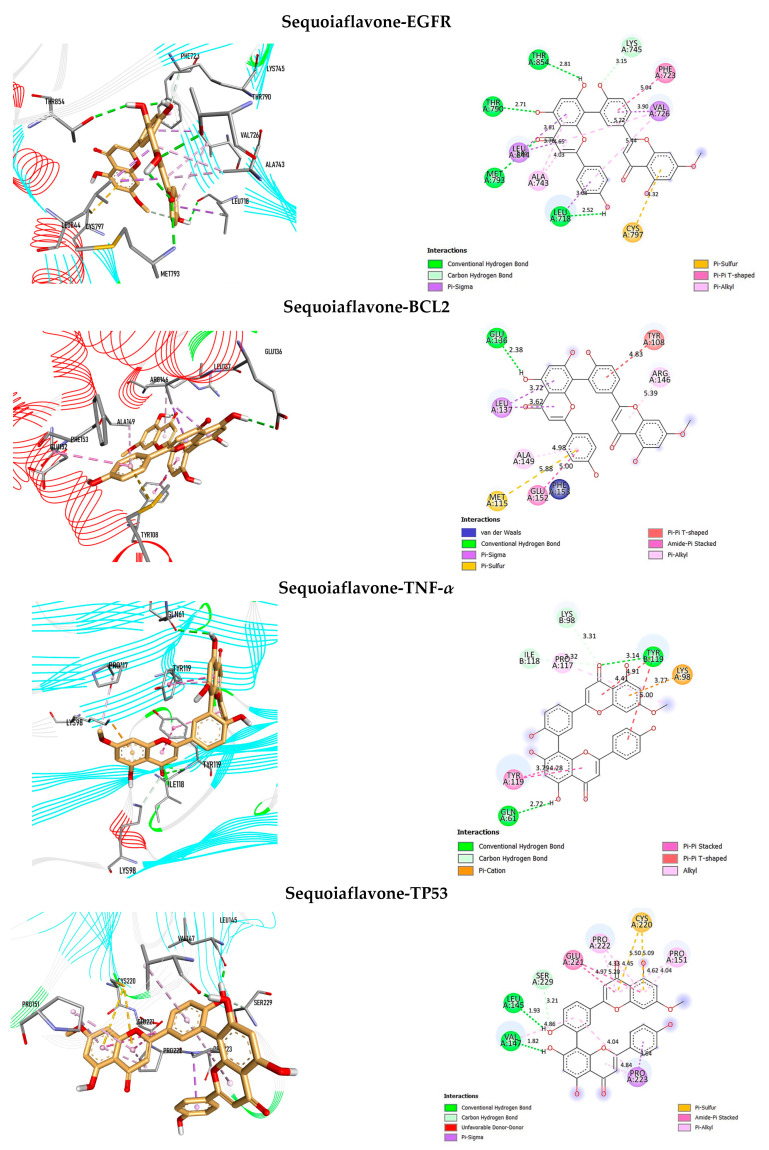

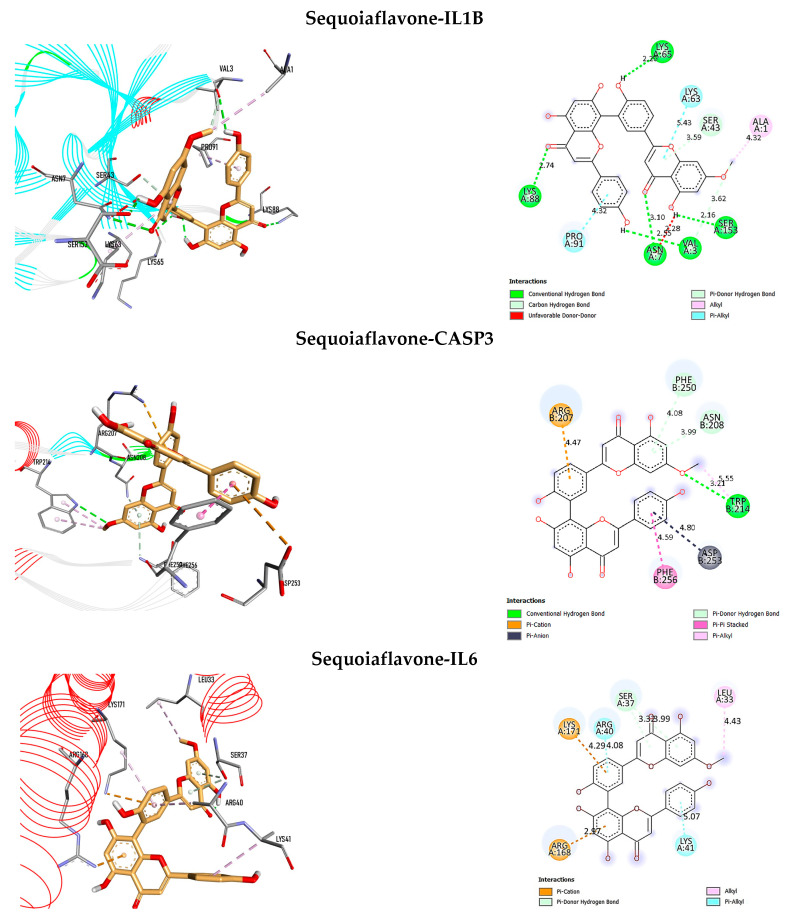
Three-dimensional and two-dimensional representations of the interaction complexes of sequoiaflavone with HSP90AA1, EGFR, BCL2, TNF-*α*, TP53, IL1B, CASP3, and IL6. The plots have been arranged in ascending order according to their respective docking score values.

**Figure 6:**
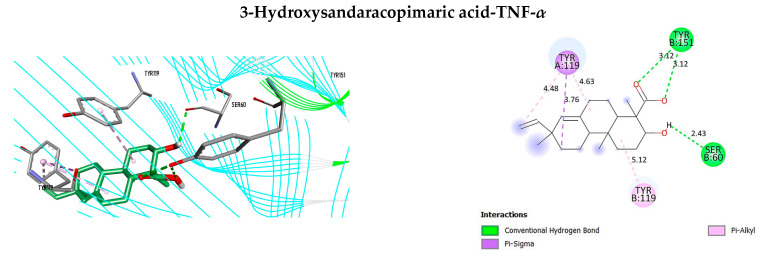

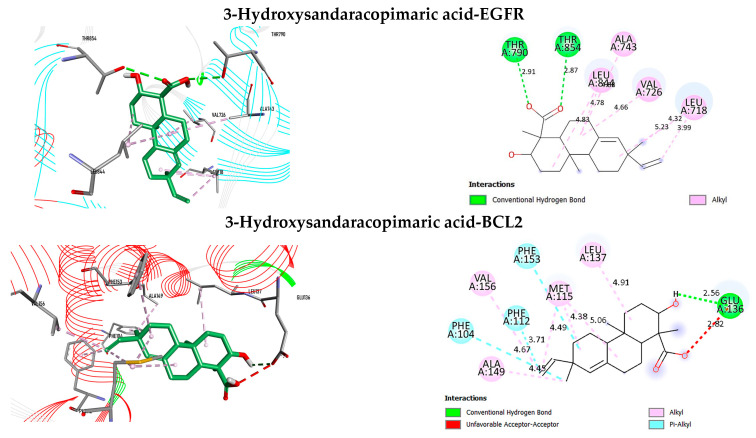
Three-dimensional and two-dimensional representations of the interaction complexes of 3-hydroxysandaracopimaric acid with TNF-*α*, EGFR, and BCL2. The plots have been arranged in ascending order according to their respective docking score values.

**Figure 7:**
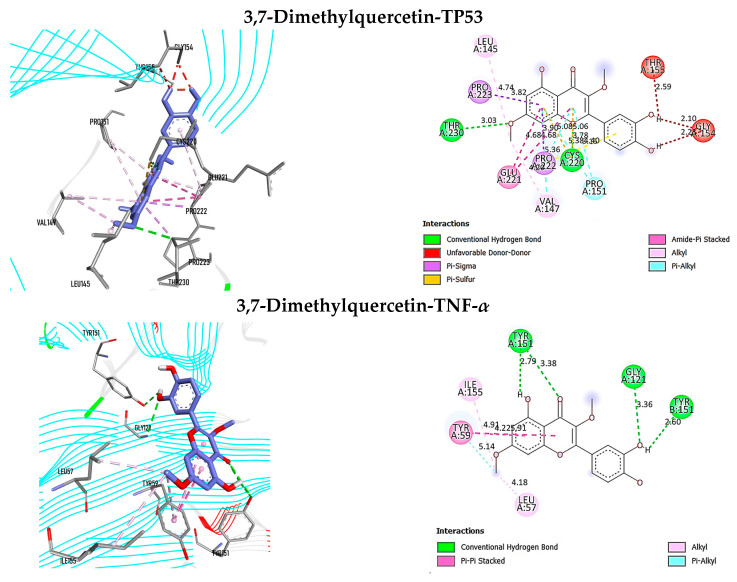

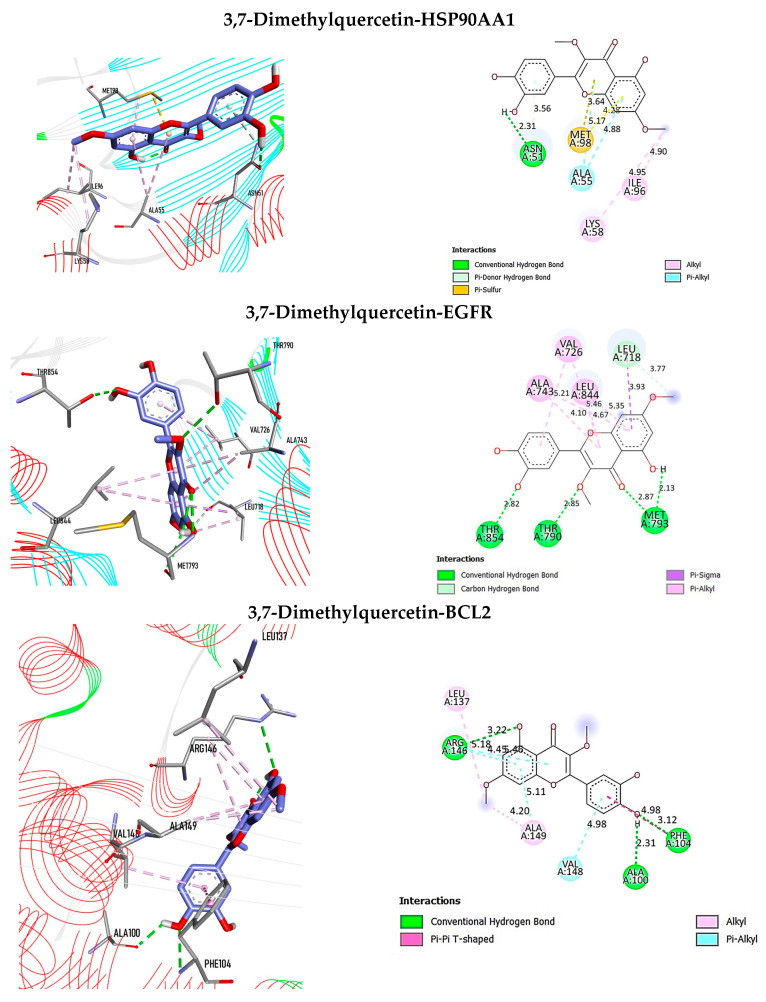
Three-dimensional and two-dimensional representations of the interaction complexes of 3,7-dimethylquercetin with TP53, TNF-*α*, HSP90AA1, EGFR, and BCL2. The plots have been arranged in ascending order according to their respective docking score values.

Table 4 displays the results of the docking analysis, which includes the docking scores, interacting amino acid residues at the active sites, and associated bond types. In accordance with Autodock Vina, a lower docking score indicates a stronger ligand–receptor association, with a score below −7 kcal/mol indicating a high binding affinity [101]. The interaction complexes with docking scores below −7 kcal/mol are illustrated in Figure 5, Figure 6 and Figure 7 organized in ascending order of score values for each ligand.

The findings revealed that sequoiaflavone exhibited the highest binding affinity for all the proteins analyzed in this study. Significantly, the most favorable results were observed with HSP90AA1, EGFR, BCL2, TNF-*α*, and TP53 exhibiting docking scores of −10.27, −10.14, −10.13, −9.429, and −9.060 kcal/mol, respectively.

As depicted in Figure 5, the interaction complex between sequoiaflavone and HSP90AA1 manifested a total of twelve intermolecular interactions. Among these, three were attributed to hydrogen bonds, wherein sequoiaflavone interacted with Ser50 and Gly97 through conventional hydrogen bonding, and with Asn51 via Pi-donor–hydrogen bond. On the other hand, the docked complex of sequoiaflavone and EGFR displayed remarkably fifteen intermolecular bonds that involved four conventional hydrogen bonds with Leu718, Thr790, Met793, and Thr854, along with one carbon–hydrogen bond with Lys745. Furthermore, it was observed that sequoiaflavone and BCL2 exhibited nine intermolecular interactions, including a single conventional hydrogen bonding with Glu136. Additionally, the interaction between sequoiaflavone and TNF-*α* is mediated by ten intermolecular linkages, including two conventional hydrogen bonds formed with GlnA61 and TyrB119, as well as two additional carbon–hydrogen bonds with LysB98 and IleB118 residues located beyond the active site. Sequoiaflavone was found to form fifteen intermolecular bonds with TP53, including two conventional hydrogen bonds with Leu145 and Val147, as well as a carbon–hydrogen bond with Ser229.

As illustrated in Figure 6, the interaction analysis revealed the presence of seven intermolecular interactions between 3-hydroxysandaracopimaric acid and TNF-*α*. Notably, three conventional hydrogen bonds were identified, with one being associated with the SerB60 residue and the remaining two with the TyrB151 residue. Moreover, the interaction between 3-hydroxysandaracopimaric acid and EGFR resulted in the formation of nine intermolecular bonds, which included two conventional hydrogen bonds that were established with Thr790 and Thr854. In addition, eleven intermolecular interactions were detected between 3-hydroxysandaracopimaric acid and BCL2, where a conventional hydrogen bond was formed with Glu136 residue.

Furthermore, it was observed that 3,7-dimethylquercetin demonstrated a significant potential in its ability to bind with TP53, TNF-α, HSP90AA1, EGFR, and BCL2. The docking scores for these interactions were −7.112, −7.258, −7.945, −7.868, and −7.394 kcal/mol, respectively. According to the findings presented in Figure 7, the compound 3,7-dimethylquercetin exhibited an interaction with TP53 through eighteen intermolecular associations, including two conventional hydrogen bonds with Cys220 and Thr230. Additionally, the interaction between 3,7-dimethylquercetin and TNF-*α* was characterized by nine intermolecular bonds, four of which were conventional hydrogen bonds with GlyA121, TyrA151, and TyrB151. As well, the intermolecular connection between 3,7-dimethylquercetin and HSP90AA1 was established through the formation of eight bonds, comprising a conventional hydrogen bond and a carbon–hydrogen bond, with the Asn51 residue. In relation to the interplay between 3,7-dimethylquercetin and EGFR, a total of twelve intermolecular connections were identified. These included four conventional hydrogen bonds with Thr790, Met793, and Thr854, as well as a carbon–hydrogen bond with Leu718. Moreover, it was observed that 3,7-dimethylquercetin exhibited intermolecular interactions with BCL2 via ten connections. Notably, two conventional hydrogen bonds were identified at the active site, specifically with Ala100 and Phe104. Additionally, a further hydrogen bond was detected with the Arg146 residue, which is situated beyond the active site.

### 3.4. In Vivo Validation

#### 3.4.1. CLAE Improved Liver Function

**Figure 8:**
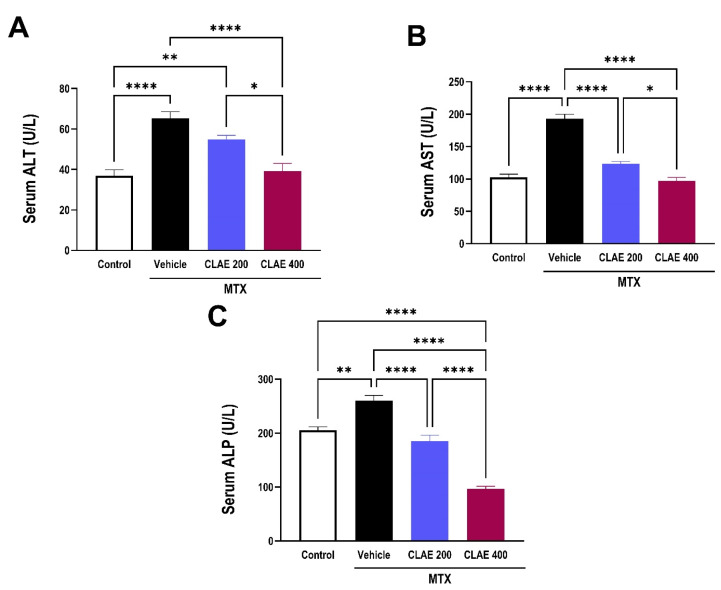
Effect of 10 days administration of *Chamaecyparis lawsoniana* extract (CLAE) at 200 and 400 mg/kg/day, gavage on impaired liver function induced by single i.p injection of methotrexate (MTX) at a dose of 20 mg/kg on the fifth day of the experiment. Liver function is presented as serum levels of alanine aminotransferase (ALT, (**A**)), aspartate aminotransferase (AST, (**B**)), and alkaline phosphatase (ALP, (**C**)). Values are presented as mean ± SEM (n = 6/group). Statistical analysis was conducted using one-way analysis of variance (ANOVA) followed by Tukey’s Post hoc test. **** *p* < 0.0001, ** *p* < 0.01, and * *p* < 0.05.

As depicted in Figure 8A–C, a significant impairment of liver function was exhibited in the vehicle-treaded MTX group, indicating liver injury, as expressed by elevated levels of circulating liver enzymes (ALT, AST, and ALP) compared to the control group. Hepatoprotective effects of CLAE at both doses were evident by the significant reductions in the circulating levels of ALT, AST, and ALP when compared to the vehicle-treated MTX group (Figure 8A,B,C, respectively). The higher dose of CLAE exhibited a more efficient improvement in liver function and hence hepatoprotection compared to the smaller one, indicating the dose-dependent effect of CLAE.

#### 3.4.2. CLAE Alleviated Hepatic Oxidative Stress

**Figure 9:**
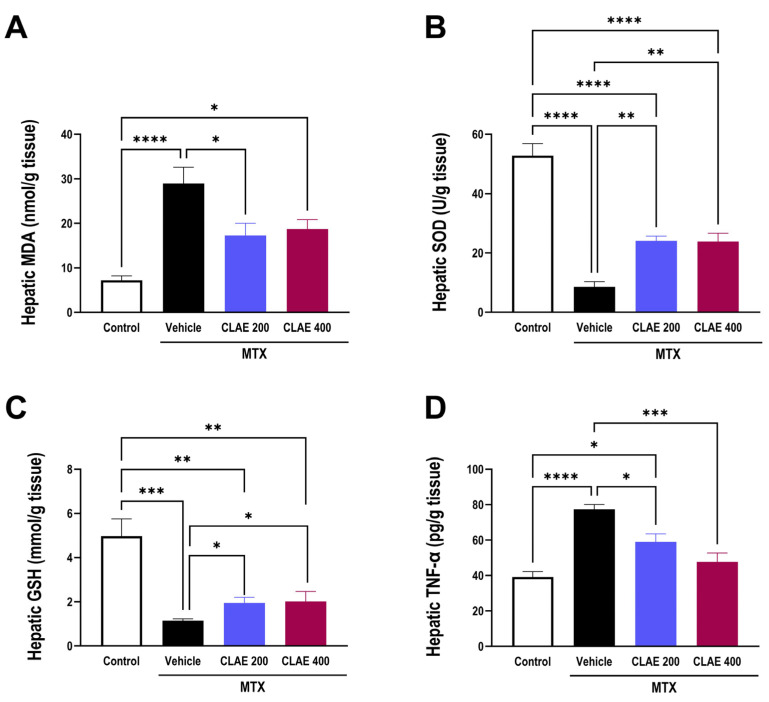
Effect of 10 days administration of *Chamaecyparis lawsoniana* extract (CLAE) at 200 and 400 mg/kg/day, gavage on hepatic oxidative stress and inflammation induced by single i.p injection of methotrexate (MTX) at a dose of 20 mg/kg on the fifth day of the experiment. Oxidative status is expressed by hepatic content of malondialdehyde (MDA, (**A**)), superoxide dismutase (SOD, (**B**)), and reduced glutathione (GSH, (**C**)). Inflammatory status is expressed by proinflammatory cytokine tumor necrosis factor-α (TNF-α, (**D**)). Values are presented as mean ± SEM (n = 6/group). Statistical analysis was conducted using one-way analysis of variance (ANOVA) followed by Tukey’s Post hoc test. **** *p* < 0.0001, *** *p* < 0.001, ** *p* < 0.01, and * *p* < 0.05.

As presented in Figure 9A–C, MTX intoxication elicited pronounced hepatic oxidative stress, as manifested by a significant increase in the lipid peroxidation product MDA and significant attenuation of the hepatic antioxidant capacity, as depicted by a decline in the SOD activity and GSH level when compared to the control group. Comparable to the vehicle-treated MTX group, both doses of CLAE significantly alleviated MTX-induced oxidative stress, where there was a significant reduction in hepatic MDA, while enhanced SOD activity and GSH level in the liver was observed upon CLAE administration, indicating the antioxidant potential of CLAE (Figure 9A–C). 

#### 3.4.3. CLAE Reduced Hepatic Inflammation

As shown in Figure 9D, the vehicle-treated MTX group exhibited significant elevation in the proinflammatory cytokine, TNF-α, indicating hepatic inflammation compared to the control group. On the other hand, CLAE significantly reduced the hepatic TNF-α content in a dose-dependent manner in comparison with the vehicle-treated MTX group.

#### 3.4.4. CLAE Attenuated Apoptosis (Immunostaining and Biochemical Findings)

**Figure 10:**
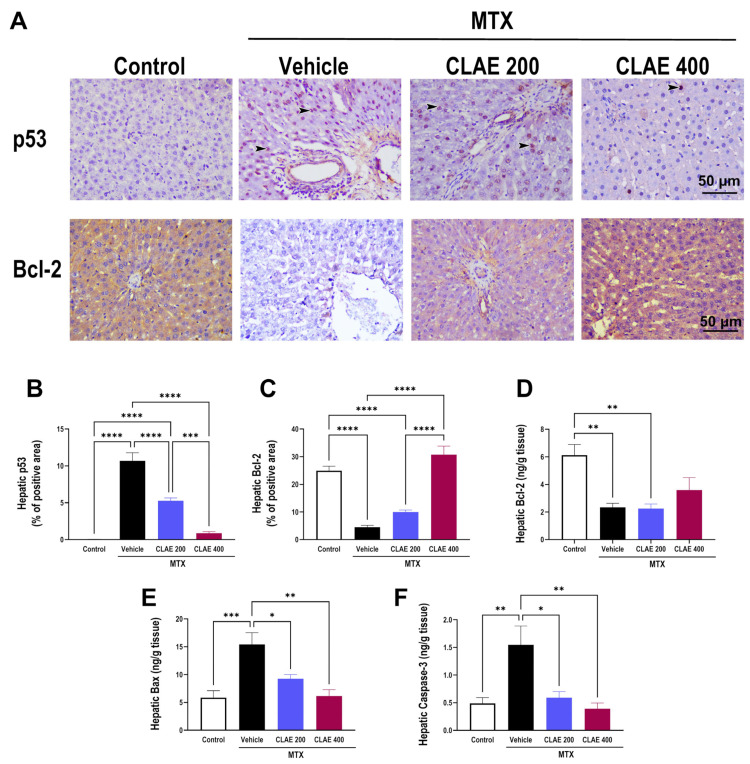
Effect of 10 days administration of *Chamaecyparis lawsoniana* extract (CLAE) at 200 and 400 mg/kg/day, gavage on hepatic apoptosis induced by single i.p injection of methotrexate (MTX) at a dose of 20 mg/kg on the fifth day of the experiment. (**A**) depicts representative micrographs of immunohistochemically stained liver sections for p53 expression (arrowhead) and Bcl-2 expression of different study groups (×400 and Scale bar, 50 μm). Positive immune reaction for the target protein is demonstrated by a brown color. (**B**,**C**) are the quantification of p53 and Bcl-2, respectively. The hepatic contents of Bcl-2 (**D**), Bax (**E**), and caspase-3 (**F**) were also shown. Values are presented as mean ± SEM (n = 6/group). Statistical analysis was conducted using one-way analysis of variance (ANOVA) followed by Tukey’s Post hoc test. **** *p* < 0.0001, *** *p* < 0.001, ** *p* < 0.01, and * *p* < 0.05.

MTX intoxication induced hepatic apoptosis, as manifested by increased positive areas of p53 staining, a proapoptotic biomarker, in hepatocyte nuclei, whereas reduced positive areas of Bcl-2-staining, antiapoptotic protein, and weak cytoplasmic immune reactivity were noticed in immunostained liver sections when compared to the control group (Figure 10A). Further, biochemical measurements revealed declined antiapoptotic Bcl-2, while the proapoptotic biomarkers Bax and caspase-3 were increased in the vehicle-treated MTX group in comparison with the control one (Figure 10B,C,D, respectively).

CLAE, in a dose-dependent manner, attenuated MTX-induced hepatic apoptosis with the remarkable downregulation of p53 immunoexpression along with the upregulation of cytosolic Bcl-2 in immunostained liver sections (Figure 10A–C). CLAE dose-dependently increased hepatic Bcl-2, while both hepatic Bax and caspase-3 (Figure 10D–F) were reduced compared to the vehicle-treated MTX group.

#### 3.4.5. CLAE Improved Liver Histology (Histopathological Findings)

**Figure 11:**
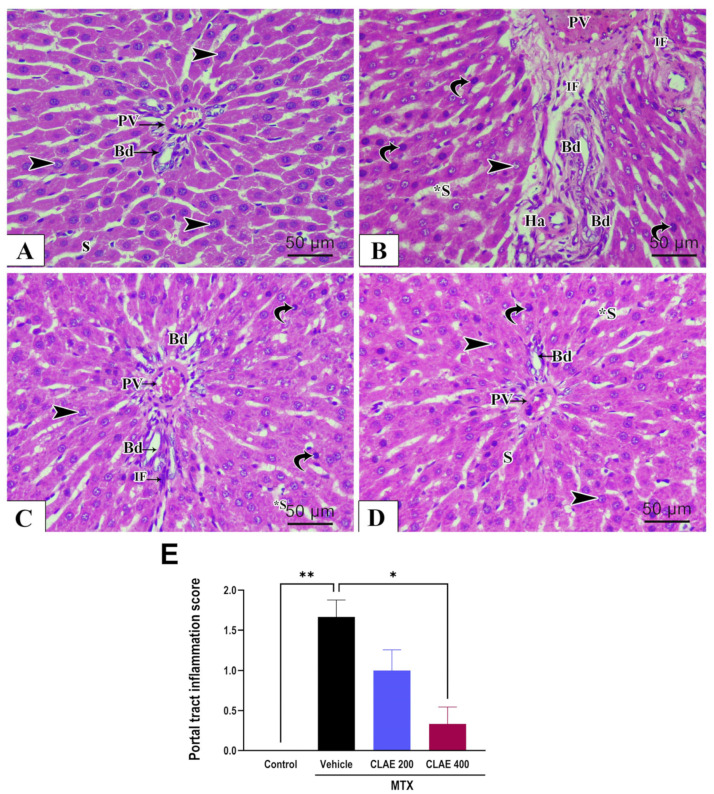
Photomicrographs of HE-stained sections of liver tissue showing histological features of different studied groups, control group (**A**), vehicle-treated methotrexate (MTX) group (**B**), *Chamaecyparis lawsoniana* extract (CLAE) at 200 and 400 mg/kg/day, gavage (**C**,**D**, respectively). Normal vesicular central nucleus (arrow), sinusoids (S), portal vein (PV), bile duct (Bd), dark pyknotic nuclei (curved arrow), dilated sinusoids (*S), inflammatory cellular infiltrations (IFs). (×400 and Scale bar, 50 μm). (**E**) shows scoring of histopathological changes in portal tract inflammatory cells. Hepatotoxicity was induced by single i.p injection of MTX at a dose of 20 mg/kg on the fifth day of the experiment, and CLAE administration started five days prior to MTX injection and continued for another 5 days. Statistical analysis for histopathological scoring was performed using Kruskal-Wallis test and Dunn’s test for multiple comparisons. ** *p* < 0.01, and * *p* < 0.05.

As displayed in Figure 11B, features of hepatopathy were observed upon the examination of H&E-stained liver sections from rats of the vehicle-treated MTX group, where most of the hepatocytes exhibited dark-stained nuclei, while few were normal. Wide separations between hepatocyte plates were depicted due to sinusoids dilatation. Inflammatory cell infiltrations close to the dilated and congested portal vein, as well as proliferated bile ductulus, were detected in the region of the portal tract. On the contrary, the control group exhibited normal hepatic architecture, where each hepatic lobule consisted of anastomosing radially distributing hepatocytes. The hepatocytes were polygonal in shape with well-defined boundaries. Their cytoplasm was acidophilic, and the majority of cells had a single rounded, vesicular, and centrally placed nucleus, whereas some cells appeared to be binucleated. The hepatic sinusoids were seen as narrow spaces in between adjacent plates of hepatocytes and lined by flat endothelial cells and Kupffer cells. The hepatic portal tracts were seen at the periphery of the lobule. Portal tracts had branches of the portal vein, hepatic artery, and bile duct (Figure 11A).

Upon examination of the liver section from rats who received the lower dose of CLAE, partial restoration of liver histological features was depicted. Some dispersed inflammatory cells through the parenchyma of the liver could be noticed. Some hepatocytes still showed dark-stained nuclei and few cellular infiltrations. Double bile ducts and dilated sinusoid could be observed (Figure 11C). Interestingly, increasing the dose of CLAE restored most of the histological features, which appear near normal patterns (Figure 11D). The vehicle-treated MTX group exhibited significantly increased portal tract inflammation scores compared to the control, while CLAE dose-dependently reduced the injury scores (Figure 11E).

## 4. Discussion

Despite the recent therapeutic advancements and significant progress in medicine, hepatic diseases continue to pose a universal health challenge. Therefore, the exploration of novel and potent drugs against liver injury is a worthwhile pursuit. While synthetic drugs have been used to treat liver diseases, they have been shown to be carcinogenic and cause severe side effects. In contrast, herbal products are cost-effective, better compatible with the human body, have lower side effects, and are easier to store. Moreover, plants are a rich source of bioactive constituents such as phenolic acids and flavonoids, making the herbal approach a viable alternative to conventional therapy [102].

Therefore, the present study focused on investigating the protective potential of *Chamaecyparis lawsoniana* aerial parts ethanolic extract (CLAE) against DILI, with a specific emphasis on liver injury caused by MTX. The research methodology was based on phytochemical profiling, which was subsequently complemented by network pharmacology and docking studies, followed by preclinical validation. By adopting the comprehensive approach, the study has successfully identified the most biologically significant components of CLAE, along with their potential molecular targets and mechanisms of action in mitigating MTX-induced liver injury.

The phytochemical profile of CLAE was investigated using UPLC–ESI–MS/MS analysis in negative mode. According to the retention time, pseuomolecular ion peak [M-H]^−^, MS^2^ fragmentation patterns, as well as the available literature, 65 phytochemicals were tentatively characterized, mainly including flavonoids, particularly bioflavonoids, and glycosides, diterpene and phenolic acids, and lignans.

Previous studies have extensively investigated the hepatoprotective effects of various components from these identified chemical classes. Flavonoids, in particular, have gained recognition for their ability to provide a substantial hepato-protective effect through diverse mechanisms. A wide range of approximately 100 bioflavonoids have been documented for their hepatoprotective activity [103]. Notably, amentoflavone, a biflavonoid, has demonstrated significant hepatoprotective activity through various mechanisms [104,105]. Moreover, significant hepatoprotective properties in diverse models of DILI have been demonstrated by other subtypes of flavonoids, specifically quercetin and its related compounds such as quercetin 7-rhamnoside, 3′-*O*-methyl quercetin, and quercetin-3-*O*-glucuronide [106,107].

Additionally, several medicinal plants containing diterpene acids, such as *Juniperus phoenicea* [108] and *Rosmarinus officinalis* [109], have been found to protect the liver from damage caused by carbon tetrachloride (CCl_4_). Additionally, extracts from *Cupressus sempervirens* leaves, rich in biflavones and phenolic acids, showed significant hepatoprotective properties against both CCl_4_-induced and paracetamol-induced damage [110,111]. *Juniperus sabina* aerial parts, containing diterpene acids, lignans, and flavonoids, also demonstrated promising hepatoprotective activity against CCl_4_-induced damage [112].

In recent years, the focus of biomedical research has shifted towards identifying pharmacological targets from active ingredients found in medicinal plants, with the ultimate goal of developing novel therapies. The emergence of network pharmacology as a systematic paradigm presents a unique opportunity to explore traditional medicines and has become a pioneering research field in drug discovery and development. This advancement has paved the way for a better understanding of the complex bioactive components found in various medicinal plants [113]. The application of the network pharmacology approach in this investigation led to the discovery of 195 significant potential targets of CLAE in DILI. Among these targets, the top eight, namely TP53, IL6, TNF-*α*, HSP90AA1, EGFR, IL1B, BCL2, and CASP3, were deemed particularly noteworthy.

Molecular docking is a computerized approach that predicts the most effective way for a ligand to attach to a receptor, forming a stable complex. It is a valuable tool for identifying potential drug targets by analyzing the binding ability of small molecules and the active pocket of the protein. A low energy complex and a compatible ligand can result in strong activity [114].

To shed light on the potential mechanisms underlying the hepatoprotective effects of CLAE against DILI, a molecular docking simulation was carried out on the three most significant bioactive compounds present in CLAE, namely sequoiaflavone, 3-hydroxysandaracopimaric acid, and 3,7-dimethylquercetin, against eight key DILI targets, including IL6, TNF-α, HSP90AA1, EGFR, IL1B, BCL2, and CASP3.

Apoptosis is a crucial intracellular process that functions as a self-destruct program, playing a pivotal role in maintaining cellular homeostasis and eliminating irreparable damaged cells [115]. Its regulation involves a complex network of genes, including TP53, which induces cell apoptosis by controlling the translocation of antiapoptotic Bcl-2 and pro-apoptotic Bax proteins. The activated p53 alters the permeability of the cell membrane, facilitating the release of cytochrome c from the mitochondria into the cytoplasm. Subsequently, this process triggers the activation of cleaved caspase3, initiating cell degradation [116]. This process holds a significant importance in the context of liver injury [117] since evidence suggested that the p53 protein accumulates in individuals with various inflammatory liver diseases. Inhibiting the p53 signaling pathway has been demonstrated to enhance drug-induced hepatocyte injury by regulating the mitochondrial apoptosis pathway. Consequently, this presents a promising therapeutic strategy for effectively treating liver injury [118]. During molecular docking, TP53 exhibited a robust binding affinity towards the CLAE components sequoiaflavone and 3,7-dimethylquercetin. Within the Bcl-2 active pocket, sequoiaflavone, 3-hydroxysandaracopimaric acid, and 3,7-dimethylquercetin displayed promising binding energies, suggesting their potential for actively contributing to the hepatoprotective effect by modulating apoptosis.

On the other hand, inflammation constitutes a significant factor in the development of drug-induced toxicities, including those caused by MTX. This is due to the generation of free radicals and associated oxidative stress, which are known to initiate inflammatory responses. As a result, proinflammatory cytokines such as TNF-*α*, IL-1β, and IL-6 are secreted, leading to tissue injury [119]. However, this study showed that the investigated CLAE constituents could have the potential to downregulate these mediators by interacting with their active sites, particularly TNF-*α*, alleviating the inflammation associated with DILI.

Furthermore, a correlation between heat shock protein 90 (HSP90) and hepatic injury was previously reported, and it was observed that HSP90 inhibitors exhibited a protective effect on various organs [120,121]. Additionally, the EGFR is implicated in the pathogenesis of both cirrhosis and hepatocellular carcinoma (HCC), with its hepatic expression increasing during cirrhosis [122]. Studies have suggested that inhibiting EGFR may offer a promising therapeutic strategy for reducing fibrogenesis and preventing HCC in patients with high-risk cirrhosis [123,124]. The results from the docking analysis revealed that sequoiaflavone and 3,7-dimethylquercetin could possess inhibitory properties against HSP90. Furthermore, these compounds also exhibited the ability to inhibit EGFR, along with 3-hydroxysandaracopimaric acid. This dual inhibition potential may play a crucial role in safeguarding the liver against hepatotoxicity.

Based on the simulation results, the compounds displayed favorable affinities for binding to the targeted proteins. It is noteworthy to highlight that sequoiaflavone exhibited an exceptionally strong binding affinity towards all the targeted proteins. These findings imply that these components might possess synergistic hepatoprotective effects through multiple mechanisms. Consequently, CLAE shows promise as a preventive approach against DILI caused by these proteins.

To achieve a comprehensive appraisal, it is essential to perform an experimental validation as this furnishes supplementary evidence and verification of the conclusions derived from computational analysis. Consequently, this study employed a preclinical model of MTX-induced liver injury in rats to investigate the potential hepatoprotective effects and underlying mechanism of action of CLAE.

In this study, MTX intoxication elicited hepatotoxicity, as manifested by significant augmentation in circulating liver function enzymes (AST, ALT, and ALP) and disrupted histological architecture, which is consistent with previous studies [125,126]. However, the administration of CLAE demonstrated hepatoprotective potential, as expressed by significant dose-dependent decrease in AST, ALT, and ALP circulating levels, and the restoration of normal hepatic histological features, where the smaller dose of CLAE elicited partial restoration, while an increasing dosage reinstated the majority of these characteristics, closely resembling normal patterns.

Ample evidence suggests that MTX-induced multiorgan injury involves oxidative stress, which is a consequence of ROS activation [10,127,128] and results in a decline in antioxidant defenses [129], which is consistent with our findings where challenging rats with MTX significantly augmented the MDA level while attenuating the GSH level and SOD activity in liver. CLAE depicted significant antioxidant potential by reducing hepatic MDA levels while enhancing the hepatic antioxidant capacity expressed as SOD activity and GSH levels, thereby alleviating MTX-induced oxidative stress. High-dose MTX-associated oxidative stress triggered the release of proinflammatory cytokines, which further contributes to tissue injury [130,131]; this supports our results where elevated hepatic TNF-*α* following MTX intoxication was found. CLAE significantly and dose-dependently reduced hepatic inflammation by reducing TNF-*α* levels. ROS overproduction during MTX therapy provokes DNA damage and triggers apoptotic pathways, as reported in several studies [126,132]. In this study, MTX upregulated p53, proapoptotic Bax, and caspase-3, while it downregulated antiapoptotic Bcl-2, thus inducing apoptotic changes, adding to MTX-induced hepatotoxicity. CLAE attenuated MTX-induced hepatic apoptosis by downregulating p53 expression while upregulating cytosolic Bcl-2, as depicted in immunostained liver sections. CLAE dose-dependently enhanced hepatic Bcl-2 while decreasing Bax and caspase-3.

Collectively, these findings highlight the potential hepatoprotective benefits of CLAE in reversing the detrimental effects of MTX-induced hepatopathy, and this effect may be attributed to one or more of its bioactive components. Further research and investigation are warranted to fully understand the mechanisms underlying this restoration and to explore the clinical implications of these findings.

## 5. Conclusions

In conclusion, our research findings, supported by comprehensive in silico and in vivo studies, present compelling evidence for the hepatoprotective properties of CLAE in DILI, with a specific focus on MTX-induced liver injury. Moreover, our investigations have elucidated the underlying mechanism of action of CLAE. Nevertheless, additional preclinical and clinical studies are imperative to assess the efficacy and safety of CLAE in DILI cases, and to evaluate any potential long-term complications that may arise.

## Figures and Tables

**Table 2:** Top common targets ranked by the Degree method.

Rank	Target Name	Score
1	TP53	59
2	IL6	50
3	TNF	46
3	HSP90AA1	46
5	EGFR	44
6	IL1B	43
7	BCL2	42
8	CASP3	37
8	JUN	37
10	ALB	36
11	MMP9	35
12	HIF1A	34
13	ESR1	30
14	PTGS2	29
15	STAT1	28
16	MAPK3	26
17	ERBB2	25
18	MAPK1	24
19	MAPK8	23
19	JAK2	23

**Table 3:** Bioactive compounds of CLAE ranked by the Degree method.

Rank	Compound	Score
1	Sequoiaflavone	105
2	3-Hydroxysandaracopimaric acid	104
2	3,7-Dimethylquercetin	104
4	12*α*-hydroxy-8,15-isopimaradien-18-oic acid	103
5	Robustaflavone 7,4′-dimethyl ether	102
6	Bilobetin	100
6	4′,5,6,7-Tetramethoxyflavone (Scutellarein tetramethyl ether)	100
8	8alpha-8-Hydroxy-12-oxo-13-abieten-18-oic acid	99
8	Carnosol	99
10	Isoginkgetin	97
11	Matairesinol	94
12	Caffeoylshikimic acid	93
13	secoisolariciresinol guaiacylglyceryl ether	92
14	*ent*-Kaurenoic acid	90
15	Ferulic acid O-glucoside	89
15	Roseoside	89
17	lariciresinol-4′-guaiacylglyceryl ether	88
17	cyclolariciresinol	88
19	Sinapoyl D-glucoside	87
19	Malyngic Acid	87
21	Copalic acid	86
21	Naringenin	86
23	Coumaroylquinic acid	82
24	Pinopalustrin (Nortrachelogenin)	80
24	Kaempferol-3-*O*-arabinoside	80
26	Aromadendrin	77
27	Quinic acid	76
28	Phlorizin	74
29	Vanillic acid glucoside	68
30	L-Threonic acid	59
31	Protocatechualdehyde	42

**Table 4:** Molecular docking results of the top three CLAE bioactive constituents against the top eight target proteins.

Target Protein	Ligand	Docking Score (kcal/mol)	Interacting Amino Acid Residues	Bond Type
TP53 (8DC4)	Sequoiaflavone	−9.060	Glu221 Ser229 Leu145 and Val147 Val147, Pro151, Pro222, and Pro223 Pro223 Cys220	Amide-Pi Stacked Carbon–Hydrogen Conventional Hydrogen *Pi*-Alkyl *Pi*-Sigma *Pi*-Sulfur
	3-Hydroxysandaracopimaric acid	−6.291	Pro151, Pro222, and Pro223 Val147	Alkyl Conventional Hydrogen
	3,7-Dimethylquercetin	−7.112	Leu145 and Val147 Glu221 Cys220 and Thr230 Val147, Pro151, and Pro222 Pro222 and Pro223 Cys220 Gly154 and Thr155	Alkyl Amide-*Pi* Stacked Conventional Hydrogen *Pi*-Alkyl *Pi*-Sigma *Pi*-Sulfur Unfavorable Donor–Donor
	Co-crystallized ligand	−7.040	Pro223 Glu221 Cys220 Val147, Pro151, Pro222, and Pro223 Thr230 Val147 Cys220	Alkyl Amide-*Pi* Stacked Conventional Hydrogen *Pi*-Alkyl *Pi*-Donor–Hydrogen *Pi*-Sigma *Pi*-Sulfur
IL6 (4NI9)	Sequoiaflavone	−7.444	Leu33 Lys41 and Arg40 Arg168 and Lys171 Ser37	Alkyl *Pi*-Alkyl *Pi*-Cation *Pi*-Donor–Hydrogen
	3-Hydroxysandaracopimaric acid	−4.837	Leu33, Arg40, and Lys171	Alkyl
	3,7-Dimethylquercetin	−6.277	Leu33 Ser37 Arg40, Arg168, and Lys171 Lys171 Ser37 Arg168	Alkyl Carbon–Hydrogen *Pi*-Alkyl *Pi*-Cation *Pi*-Donor–Hydrogen Unfavorable Donor–Donor
* TNF-*α* (2AZ5)	Sequoiaflavone	−9.429	ProA117 LysB98 and IleB118 GlnA61 and TyrB119 LysA98 TyrA119 TyrB119	Alkyl Carbon–Hydrogen Conventional Hydrogen *Pi*-Cation *Pi*-*Pi* Stacked *Pi*-*Pi* T-shaped
	3-Hydroxysandaracopimaric acid	−8.56	SerB60 and TyrB151 TyrA119 and TyrB119 TyrA119	Conventional Hydrogen *Pi*-Alkyl *Pi*-Sigma
	3,7-Dimethylquercetin	−7.258	LeuA57 and IleA155 GlyA121, TyrA151, and TyrB151 TyrA59 TyrA59	Alkyl Conventional Hydrogen *Pi*-Alkyl *Pi*-*Pi* Stacked
	Co-crystallized ligand	−9.076	GlyA121 TyrB59, TyrB119, and TyrB151 TyrA119	Halogen (Fluorine) *Pi*-Alkyl *Pi*-Sigma
HSP90AA1 (8AGI)	Sequoiaflavone	−10.27	Asn51 Ser50 and Gly97 Ala55, Met98, and Val 168 Asp54 Asn51 Met98 Ser52	Amide-*Pi* Stacked Conventional Hydrogen *Pi*-Alkyl *Pi*-Anion *Pi*-Donor–Hydrogen *Pi*-Sigma Van Der Waals
	3-Hydroxysandaracopimaric acid	−6.905	Ala55, Lys58, and Met98 Gly132 Gly132 Gly135	Alkyl Conventional Hydrogen Unfavorable Acceptor–Acceptor Carbon–Hydrogen
	3,7-Dimethylquercetin	−7.945	Lys58 and Ile96 Asn51 Asn51 Ala55 and Met98 Met98	Alky Carbon–Hydrogen Conventional Hydrogen *Pi*-Alkyl *Pi*-Sulfur
	Co-crystallized ligand	−9.931	Ile96, Met98, and Leu107 Asp93, Gly97, Asn106, and Thr184 Phe138 Ala55 Met98	Alkyl Conventional Hydrogen *Pi*-Alkyl *Pi*-Sigma *Pi*-Sulfur
EGFR (7T4I)	Sequoiaflavone	−10.14	Lys745 Leu718, Thr790, Met793, and Thr854 Val726 and Ala743 Leu718, Val726, and Leu844 Cys797 Phe723	Carbon–Hydrogen Conventional Hydrogen *Pi*-Alkyl *Pi*-Sigma *Pi*-Sulfur *Pi*-*Pi* T-shaped
	3-Hydroxysandaracopimaric acid	−8.331	Leu718, Val726, Ala743, and Leu844 Thr790 and Thr854	Alkyl Conventional Hydrogen
	3,7-Dimethylquercetin	−7.868	Leu718 Thr790, Met793, and Thr854 Val726, Ala743, and Leu844 Leu718	Carbon–Hydrogen Conventional Hydrogen *Pi*-Alkyl *Pi*-Sigma
	Co-crystallized ligand	−9.079	Leu718, Val726, Ala743, Lys745, and Leu792 Asp800 and Glu804 Leu718, Gln791, and Asp800 Thr790, Met793, Phe795, Cys797, and Thr854 Val726 and Ala743 Leu718, Val726, and Leu844	Alkyl Attractive Charge Carbon–Hydrogen Conventional Hydrogen *Pi*-Alkyl *Pi*-Sigma
IL1B (1T4Q)	Sequoiaflavone	−8.833	Ala1 Val3 Val3, Asn7, Lys65, Lys88, and Ser153 Lys63 and Pro91 Ser43 Asn7	Alkyl Carbon–Hydrogen Conventional Hydrogen *Pi*-Alkyl *Pi*-Donor–Hydrogen Unfavorable Donor–Donor
	3-Hydroxysandaracopimaric acid	−6.477	Ser5 Ser43 Tyr68	Carbon–Hydrogen Conventional Hydrogen *Pi*-Alkyl
	3,7-Dimethylquercetin	−6.588	Pro87 Ser43, Glu64, Leu62, and Lys65 Pro91 Val3 Ser5	Alkyl Conventional Hydrogen *Pi*-Alkyl Unfavorable Acceptor–Acceptor Unfavorable Donor–Donor
BCL2 (7LHB)	Sequoiaflavone	−10.13	Glu152 Glu136 Arg146 and Ala149 Tyr108 Leu137 Met115 Phe153	Amide *Pi*-Stacked Conventional Hydrogen *Pi*-Alkyl *Pi*-*Pi* T-shaped *Pi*-Sigma *Pi*-Sulfur Van Der Waals
	3-Hydroxysandaracopimaric acid	−7.917	Met115, Leu137, Ala149, and Val156 Glu136 Phe104, Phe112, and Phe153 Glu136	Alkyl Conventional Hydrogen *Pi*-Alkyl Unfavorable Acceptor–Acceptor
	3,7-Dimethylquercetin	−7.394	Leu137 and Ala149 Ala100, Phe104, and Arg146 Arg146, Val148, and Ala149 Phe104	Alkyl Conventional Hydrogen *Pi*-Alkyl *Pi*-*Pi* T-shaped
	Co-crystallized ligand	−12.78	Ala100, Val133, Leu137, and Val156 Gly145 Arg107 and Asp111 Ala100, Asp103, and Asp111 Asp103 and Asn143 Glu152 Ala100, Phe112, Met115, Arg146, Val148, and Ala149 Tyr202 Tyr202	Alkyl Amide *Pi*-Stacked Attractive Charge Carbon–Hydrogen Conventional Hydrogen Halogen (Cl, Br, I) *Pi*-Alkyl *Pi*-Donor–Hydrogen *Pi*-*Pi* Stacked
CASP3 (3KJF)	Sequoiaflavone	−8.477	Trp214 Trp214 Asp253 Arg207 Asn208 and Phe250 Phe256	Conventional Hydrogen *Pi*-Alkyl *Pi*-Anion *Pi*-Cation *Pi*-Donor–Hydrogen *Pi*-*Pi* Stacked
	3-Hydroxysandaracopimaric acid	−6.334	Phe250 Asn208 and Phe250 Phe250	Carbon–Hydrogen Conventional Hydrogen *Pi*-Alkyl
	3,7-Dimethylquercetin	−6.261	Arg207 and Ser251 Phe256 Trp206 Trp214	Conventional Hydrogen *Pi*-Alkyl *Pi*-*Pi* T-shaped Unfavorable Donor–Donor
	Co-crystallized ligand	−8.20	Arg207 Arg207, Asn208, Ser209, Trp214, and Phe250 Arg207, Asn208, and Ser251 Phe250 and Phe252 Phe256	Attractive Charge Conventional Hydrogen Carbon–Hydrogen *Pi*-Alkyl *Pi*-*Pi* Stacked/3.72

* The TNF-*α* model is based on the co-crystal structure of the TNF-*α* dimer.

## Data Availability

All data and materials used are available in the manuscript and Supplementary Materials.

## Supplementary Materials

The following supporting information can be downloaded at: https://www.mdpi.com/article/10.3390/antiox12122118/s1, Figure S1: UPLC-ESI-MS/MS total ion chromatograms of CLAE in negative ion mode; Figure S2: CLAE compounds-DILI targets network; Table S1: Target proteins, the corresponding grid coordinates, and amino acid residues of the active sites; Table S2: Pharmacokinetics and the drug-likeness properties of CLAE constituents; Table S3: Bioactive compounds of CLAE; Table S4: Molecular targets of CLAE bioactive compounds; Table S5: Molecular targets associated with DILI; Table S6: Molecular targets of CLAE associated with DILI; Table S7: Core genes in PPI network ranked by the Degree method; Table S8: Detailed information of GO analysis for biological processes; Table S9: Detailed information of GO analysis for cellular components; Table S10: Detailed information of GO analysis for molecular functions; Table S11: Detailed information of KEGGs pathway analysis.

## References

[1] Yang Y., Zhou K., Ma M., Liu H., Jin M., Yin C., Wang S., Zhang J. (2023). Thiol “click” chromene mediated cascade reaction Forming coumarin for in-situ imaging of thiol flux in drug-induced liver injury. Chem. Eng. J..

[2] Parthasarathy M., Prince S.E. (2023). Andrographis paniculata (Burm. f.) Nees Alleviates Methotrexate-Induced Hepatotoxicity in Wistar Albino Rats. Life.

[3] Pivovarov K., Zipursky J.S. (2019). Low-dose methotrexate toxicity. CMAJ.

[4] Hamed K.M., Dighriri I.M., Baomar A.F., Alharthy B.T., Alenazi F.E., Alali G.H., Alenazy R.H., Alhumaidi N.T., Alhulayfi D.H., Alotaibi Y.B. (2022). Overview of Methotrexate Toxicity: A Comprehensive Literature Review. Cureus.

[5] Koźmiński P., Halik P.K., Chesori R., Gniazdowska E. (2020). Overview of dual-acting drug methotrexate in different neurological diseases, autoimmune pathologies and cancers. Int. J. Mol. Sci..

[6] Hagner N., Joerger M. (2010). Cancer chemotherapy: Targeting folic acid synthesis. Cancer Manag. Res..

[7] Shea B., Swinden M.V., Ghogomu E.T., Ortiz Z., Katchamart W., Rader T., Bombardier C., Wells G.A., Tugwell P. (2013). Folic acid and folinic acid for reducing side effects in patients receiving methotrexate for rheumatoid arthritis. Cochrane Database Syst. Rev..

[8] Shahani S., Mehraban N., Talebpour Amiri F., Abedi S.M., Noaparast Z., Mohammadinia S. (2023). Melissa Officinalis L. aqueous extract pretreatment decreases methotrexate-induced hepatotoxicity at lower dose and increases 99mTc-phytate liver uptake, as a probe of liver toxicity assessment, in rats. Ann. Nucl. Med..

[9] Herman S., Zurgil N., Deutsch M. (2005). Low dose methotrexate induces apoptosis with reactive oxygen species involvement in T lymphocytic cell lines to a greater extent than in monocytic lines. Inflamm. Res..

[10] Abbas N.A., El-Sayed S.S., El-Fatah A., Salah S., Sarhan W.M., Sarhan O., Abdelghany E., Mahmoud S.S. (2023). Losartan prevents methotrexate-induced liver and lung injury in rats via targeting PPAR-γ/TGF-β1/SMAD3 and Nrf2/redox signaling. Zagazig Univ. Med. J..

[11] Tunalı-Akbay T., Sehirli O., Ercan F., Sener G. (2010). Resveratrol protects against methotrexate-induced hepatic injury in rats. J. Pharm. Pharm. Sci..

[12] Çaglar Y., Özgür H., Matur I., Yenilmez E.D., Tuli A., Gönlüsen G., Polat S. (2013). Ultrastructural evaluation of the effect of N-acetylcysteine on methotrexate nephrotoxicity in rats. Histol. Histopathol..

[13] Al Maruf A., O’Brien P.J., Naserzadeh P., Fathian R., Salimi A., Pourahmad J. (2018). Methotrexate induced mitochondrial injury and cytochrome c release in rat liver hepatocytes. Drug Chem. Toxicol..

[14] Jahovic N., Çevik H., Şehirli A.Ö., Yeğen B.Ç., Şener G. (2003). Melatonin prevents methotrexate-induced hepatorenal oxidative injury in rats. J. Pineal Res..

[15] Ali N., Rashid S., Nafees S., Hasan S.K., Shahid A., Majed F., Sultana S. (2017). Protective effect of Chlorogenic acid against methotrexate induced oxidative stress, inflammation and apoptosis in rat liver: An experimental approach. Chem. Biol. Interact..

[16] Elmore S. (2007). Apoptosis: A review of programmed cell death. Toxicol. Pathol..

[17] Pawlak A., Kutkowska J., Obmińska-Mrukowicz B., Rapak A. (2017). Methotrexate induces high level of apoptosis in canine lymphoma/leukemia cell lines. Res. Vet. Sci..

[18] Wei C.W., Yu Y.L., Chen Y.H., Hung Y.T., Yiang G.T. (2019). Anticancer effects of methotrexate in combination with α-tocopherol and α-tocopherol succinate on triple-negative breast cancer. Oncol. Rep..

[19] Xiong S., Song D., Xiang Y., Li Y., Zhong Y., Li H., Zhang P., Zhou W., Zeng X., Zhang X. (2020). Reactive oxygen species, not Ca2+, mediates methotrexate-induced autophagy and apoptosis in spermatocyte cell line. Basic Clin. Pharmacol. Toxicol..

[20] Choy E.H.S., Smith C., Doré; C.J., Scott D.L. (2005). A meta-analysis of the efficacy and toxicity of combining disease-modifying anti-rheumatic drugs in rheumatoid arthritis based on patient withdrawal. Rheumatology.

[21] Manna K., Khan Z.S., Saha M., Mishra S., Gaikwad N., Bhakta J.N., Banerjee K., Das Saha K. (2023). Manjari Medika Grape Seed Extract Protects Methotrexate-Induced Hepatic Inflammation: Involvement of NF-κB/NLRP3 and Nrf2/HO-1 Signaling System. J. Inflamm. Res..

[22] Li L., Ju J., Zhuang X., Li S., Ma R., Li J., Ding M., Ma C., Wang X., Zhang B. (2023). Chemistry of Bairui granules and its mechanisms in the protective effect against methotrexate-induced liver injury. Phytomedicine.

[23] Azadian R., Mohammadalipour A., Memarzadeh M.R., Hashemnia M., Aarabi M.H. (2023). Examining hepatoprotective effects of astaxanthin against methotrexate-induced hepatotoxicity in rats through modulation of Nrf2/HO-1 pathway genes. Naunyn-Schmiedeberg’s Arch. Pharmacol..

[24] Sharifi F., Jazi V., Assadi Soumeh E. (2023). Elecampane rhizome extract alleviates methotrexate-induced hepatotoxicity and nephrotoxicity in male rats. Adv. Tradit. Med..

[25] Houston Durrant T., Caudullo G. (2016). Chamaecyparis Lawsoniana in Europe: Distribution, Habitat, Usage and Threats.

[26] Rathi A., Baburaj D., Sundaram E., Kumar S., Khurana A., Manchanda R. (2015). Pharmacognostic study of *Chamaecyparis lawsoniana* (Murr.) Parl.: A drug used in Homoeopathy. Indian J. Res. Homoeopath..

[27] Zazharskyi V., Davydenko P., Kulishenko O., Borovik I., Kabar A., Brygadyrenko V. (2020). Antibacterial and fungicidal effect of ethanol extracts from *Juniperus sabina*, *Chamaecyparis lawsoniana*, *Pseudotsuga menziesii* and *Cephalotaxus harringtonia*. Regul. Mech. Biosyst..

[28] Gao H., Shupe T.F., Hse C.Y., Eberhardt T.L. (2006). Antioxidant activity of extracts from the bark of *Chamaecyparis lawsoniana* (A. Murray) Parl. Holzforschung.

[29] Mohamed M.E., Tawfeek N., Elbaramawi S.S., Elbatreek M.H., Fikry E. (2022). Agathis robusta Bark Extract Protects from Renal Ischemia-Reperfusion Injury: Phytochemical, In Silico and In Vivo Studies. Pharmaceuticals.

[30] Daina A., Michielin O., Zoete V. (2017). SwissADME: A free web tool to evaluate pharmacokinetics, drug-likeness and medicinal chemistry friendliness of small molecules. Sci. Rep..

[31] Wang X., Shen Y., Wang S., Li S., Zhang W., Liu X., Lai L., Pei J., Li H. (2017). PharmMapper 2017 update: A web server for potential drug target identification with a comprehensive target pharmacophore database. Nucleic Acids Res..

[32] Daina A., Michielin O., Zoete V. (2019). SwissTargetPrediction: Updated data and new features for efficient prediction of protein targets of small molecules. Nucleic Acids Res..

[33] Zaru R., Orchard S. (2023). UniProt Tools: BLAST, Align, Peptide Search, and ID Mapping. Curr. Protoc..

[34] Rebhan M., Chalifa-Caspi V., Prilusky J., Lancet D. (1997). GeneCards: Integrating information about genes, proteins and diseases. Trends Genet..

[35] Safran M., Dalah I., Alexander J., Rosen N., Iny Stein T., Shmoish M., Nativ N., Bahir I., Doniger T., Krug H. (2010). GeneCards Version 3: The human gene integrator. Database.

[36] Piñero J., Ramírez-Anguita J.M., Saüch-Pitarch J., Ronzano F., Centeno E., Sanz F., Furlong L.I. (2020). The DisGeNET knowledge platform for disease genomics: 2019 update. Nucleic Acids Res..

[37] Hamosh A., Scott A.F., Amberger J.S., Bocchini C.A., McKusick V.A. (2005). Online Mendelian Inheritance in Man (OMIM), a knowledgebase of human genes and genetic disorders. Nucleic Acids Res..

[38] Szklarczyk D., Kirsch R., Koutrouli M., Nastou K., Mehryary F., Hachilif R., Gable A.L., Fang T., Doncheva N.T., Pyysalo S. (2023). The STRING database in 2023: Protein-protein association networks and functional enrichment analyses for any sequenced genome of interest. Nucleic Acids Res..

[39] Shannon P., Markiel A., Ozier O., Baliga N.S., Wang J.T., Ramage D., Amin N., Schwikowski B., Ideker T. (2003). Cytoscape: A software environment for integrated models of biomolecular interaction networks. Genome Res..

[40] Chin C.H., Chen S.H., Wu H.H., Ho C.W., Ko M.T., Lin C.Y. (2014). cytoHubba: Identifying hub objects and sub-networks from complex interactome. BMC Syst. Biol..

[41] Dennis G., Sherman B.T., Hosack D.A., Yang J., Gao W., Lane H.C., Lempicki R.A. (2003). DAVID: Database for Annotation, Visualization, and Integrated Discovery. Genome Biol..

[42] Guiley K.Z., Shokat K.M. (2023). A small molecule reacts with the p53 somatic mutant Y220C to rescue wild-type thermal stability. Cancer Discov..

[43] Gelinas A.D., Davies D.R., Edwards T.E., Rohloff J.C., Carter J.D., Zhang C., Gupta S., Ishikawa Y., Hirota M., Nakaishi Y. (2014). Crystal structure of interleukin-6 in complex with a modified nucleic acid ligand. J. Biol. Chem..

[44] He M.M., Smith A.S., Oslob J.D., Flanagan W.M., Braisted A.C., Whitty A., Cancilla M.T., Wang J., Lugovskoy A.A., Yoburn J.C. (2005). Small-molecule inhibition of TNF-α. Science.

[45] Tassone G., Mazzorana M., Mangani S., Petricci E., Cini E., Giannini G., Pozzi C., Maramai S. (2022). Structural Characterization of Human Heat Shock Protein 90 N-Terminal Domain and Its Variants K112R and K112A in Complex with a Potent 1, 2, 3-Triazole-Based Inhibitor. Int. J. Mol. Sci..

[46] Huang W.-S., Li F., Gong Y., Zhang Y., Youngsaye W., Xu Y., Zhu X., Greenfield M.T., Kohlmann A., Taslimi P.M. (2023). Discovery of mobocertinib, a potent, oral inhibitor of EGFR exon 20 insertion mutations in non–small cell lung cancer. Bioorganic Med. Chem. Lett..

[47] Adamek D., Guerrero L., Blaber M., Caspar D. (2005). Structural and energetic consequences of mutations in a solvated hydrophobic cavity. J. Mol. Biol..

[48] Salem A.H., Tao Z.-F., Bueno O.F., Chen J., Chen S., Edalji R., Elmore S.W., Fournier K.M., Harper K.C., Hong R. (2021). Expanding the repertoire for “Large small molecules”: Prodrug abbv-167 efficiently converts to venetoclax with reduced food effect in healthy volunteers. Mol. Cancer Ther..

[49] Wang Z., Watt W., Brooks N.A., Harris M.S., Urban J., Boatman D., McMillan M., Kahn M., Heinrikson R.L., Finzel B.C. (2010). Kinetic and structural characterization of caspase-3 and caspase-8 inhibition by a novel class of irreversible inhibitors. Biochim. Biophys. Acta (BBA)-Proteins Proteom..

[50] Burley S.K., Berman H.M., Kleywegt G.J., Markley J.L., Nakamura H., Velankar S. (2017). Protein Data Bank (PDB): The Single Global Macromolecular Structure Archive. Methods Mol. Biol..

[51] Biovia D.S. (2021). Discovery Studio Visualizer, v21. 1.0. 20298.

[52] Pettersen E.F., Goddard T.D., Huang C.C., Couch G.S., Greenblatt D.M., Meng E.C., Ferrin T.E. (2004). UCSF Chimera—A visualization system for exploratory research and analysis. J. Comput. Chem..

[53] O’Boyle N.M., Banck M., James C.A., Morley C., Vandermeersch T., Hutchison G.R. (2011). Open Babel: An open chemical toolbox. J. Cheminform..

[54] Tian W., Chen C., Lei X., Zhao J., Liang J. (2018). CASTp 3.0: Computed atlas of surface topography of proteins. Nucleic Acids Res..

[55] Mohamed A.S., Hosney M., Bassiony H., Hassanein S.S., Soliman A.M., Fahmy S.R., Gaafar K. (2020). Sodium pentobarbital dosages for exsanguination affect biochemical, molecular and histological measurements in rats. Sci. Rep..

[56] Varghese F., Bukhari A.B., Malhotra R., De A. (2014). IHC Profiler: An open source plugin for the quantitative evaluation and automated scoring of immunohistochemistry images of human tissue samples. PLoS ONE.

[57] Reel B., Guzeloglu M., Bagriyanik A., Atmaca S., Aykut K., Albayrak G., Hazan E. (2013). The effects of PPAR-γ agonist pioglitazone on renal ischemia/reperfusion injury in rats. J. Surg. Res..

[58] Al Kadhi O., Melchini A., Mithen R., Saha S. (2017). Development of a LC-MS/MS method for the simultaneous detection of tricarboxylic acid cycle intermediates in a range of biological matrices. J. Anal. Methods Chem..

[59] Fischer K., Höffler S., Meyer A. (2011). ESI single quadrupole mass spectrometric investigation of polyhydroxy acids. Anal. Lett..

[60] Cádiz-Gurrea M.L., Fernández-Ochoa Á., Leyva-Jiménez F.J., Guerrero-Muñoz N., Villegas-Aguilar M.D.C., Pimentel-Moral S., Ramos-Escudero F., Segura-Carretero A. (2020). LC-MS and Spectrophotometric Approaches for Evaluation of Bioactive Compounds from Peru Cocoa By-Products for Commercial Applications. Molecules.

[61] Zhang X., Su M., Du J., Zhou H., Li X., Zhang M., Hu Y., Ye Z. (2023). Profiling of naturally occurring proanthocyanidins and other phenolic compounds in a diverse peach germplasm by LC-MS/MS. Food Chem..

[62] Saeed M.M., Fernández-Ochoa Á., Saber F.R., Sayed R.H., Cádiz-Gurrea M.d.l.L., Elmotayam A.K., Leyva-Jiménez F.J., Segura-Carretero A., Nadeem R.I. (2022). The Potential Neuroprotective Effect of Cyperus esculentus L. Extract in Scopolamine-Induced Cognitive Impairment in Rats: Extensive Biological and Metabolomics Approaches. Molecules.

[63] Fernández-Poyatos M.d.P., Llorent-Martínez E.J., Ruiz-Medina A. (2021). Phytochemical composition and antioxidant activity of Portulaca oleracea: Influence of the steaming cooking process. Foods.

[64] Mekam P.N., Martini S., Nguefack J., Tagliazucchi D., Stefani E. (2019). Phenolic compounds profile of water and ethanol extracts of Euphorbia hirta L. leaves showing antioxidant and antifungal properties. S. Afr. J. Bot..

[65] Rodríguez-Rivera M.P., Lugo-Cervantes E., Winterhalter P., Jerz G. (2014). Metabolite profiling of polyphenols in peels of Citrus limetta Risso by combination of preparative high-speed countercurrent chromatography and LC–ESI–MS/MS. Food Chem..

[66] Ali A., Cottrell J.J., Dunshea F.R. (2023). Antioxidant, Alpha-Glucosidase Inhibition Activities, In Silico Molecular Docking and Pharmacokinetics Study of Phenolic Compounds from Native Australian Fruits and Spices. Antioxidants.

[67] Chang Chen J. (2017). A Hexa-Herbal Chinese Formula for Treatment of Atopic Dermatitis: Phytochemical Analysis and Selected Anti-Inflammatory Activities. Ph.D. Thesis.

[68] El-sayed M., Abbas F.A., Refaat S., El-Shafae A.M., Fikry E. (2021). UPLC-ESI-MS/MS Profile of The Ethyl Acetate Fraction of Aerial Parts of Bougainvillea’Scarlett O’Hara’Cultivated in Egypt. Egypt. J. Chem..

[69] Zhang Y., Xiong H., Xu X., Xue X., Liu M., Xu S., Liu H., Gao Y., Zhang H., Li X. (2018). Compounds identification in semen cuscutae by ultra-high-performance liquid chromatography (UPLCs) coupled to electrospray ionization mass spectrometry. Molecules.

[70] Petreska J., Stefkov G., Kulevanova S., Alipieva K., Bankova V., Stefova M. (2011). Phenolic compounds of mountain tea from the Balkans: LC/DAD/ESI/MSn profile and content. Nat. Prod. Commun..

[71] Ahmed R., Elkhrisy E., EL-kashak W.A.H., El Raey M., Nassar M., Aboutabl E.-S.A. (2019). Structural characterization of polyphenolics in Livistona chinensis using HPLC-PDA-MS. J. Adv. Pharm. Res..

[72] Tawfeek N., Sobeh M., Hamdan D.I., Farrag N., Roxo M., El-Shazly A.M., Wink M. (2019). Phenolic compounds from *Populus alba* L. and *Salix subserrata* Willd. (Salicaceae) counteract oxidative stress in *Caenorhabditis elegans*. Molecules.

[73] Püssa T., Pällin R., Raudsepp P., Soidla R., Rei M. (2008). Inhibition of lipid oxidation and dynamics of polyphenol content in mechanically deboned meat supplemented with sea buckthorn (*Hippophae rhamnoides*) berry residues. Food Chem..

[74] Saber F.R., Mohsen E., El-Hawary S., Eltanany B.M., Elimam H., Sobeh M., Elmotayam A.K. (2021). Chemometric-enhanced metabolic profiling of five Pinus species using HPLC-MS/MS spectrometry: Correlation to in vitro anti-aging, anti-Alzheimer and antidiabetic activities. J. Chromatogr. B.

[75] Ilina T., Kashpur N., Granica S., Bazylko A., Shinkovenko I., Kovalyova A., Goryacha O., Koshovyi O. (2019). Phytochemical profiles and in vitro immunomodulatory activity of ethanolic extracts from *Galium aparine* L.. Plants.

[76] Singh A.P., Wang Y., Olson R.M., Luthria D., Banuelos G.S., Pasakdee S., Vorsa N., Wilson T. (2017). LC-MS-MS analysis and the antioxidant activity of flavonoids from eggplant skins grown in organic and conventional environments. Food Nutr. Sci..

[77] Gulsoy-Toplan G., Goger F., Yildiz-Pekoz A., Gibbons S., Sariyar G., Mat A. (2018). Chemical constituents of the different parts of Colchicum micranthum and C. chalcedonicum and their cytotoxic activities. Nat. Prod. Commun..

[78] Smirnov K. (2018). Ultra-High Resolution Mass Spectrometry in Characterizing the Impact of Whole Grain Diet on Human Gut Meta-Metabolome. Ph.D. Thesis.

[79] Hamdan D.I., Tawfeek N., El-Shiekh R.A., Khalil H.M., Mahmoud M.Y., Bakr A.F., Zaafar D., Farrag N., Wink M., El-Shazly A.M. (2022). Salix subserrata bark extract-loaded chitosan nanoparticles attenuate neurotoxicity induced by sodium arsenate in rats in relation with HPLC–PDA-ESI–MS/MS profile. AAPS PharmSciTech.

[80] Hamany Djande C.Y., Steenkamp P.A., Piater L.A., Tugizimana F., Dubery I.A. (2022). Hordatines and associated precursors dominate metabolite profiles of barley (*Hordeum vulgare* L.) seedlings: A metabolomics study of five cultivars. Metabolites.

[81] Elnaggar D. (2022). Hypouricemic effect of *Antidesma bunius* (L.) leaves extract and identification of flavonoid content. Al-Azhar J. Pharm. Sci..

[82] Brito A., Ramirez J.E., Areche C., Sepúlveda B., Simirgiotis M.J. (2014). HPLC-UV-MS profiles of phenolic compounds and antioxidant activity of fruits from three citrus species consumed in Northern Chile. Molecules.

[83] Abdelaziz S., Al Yousef H.M., Al-Qahtani A.S., Hassan W.H., Fantoukh O.I., El-Sayed M.A. (2020). Phytochemical profile, antioxidant and cytotoxic potential of Parkinsonia aculeata L. growing in Saudi Arabia. Saudi Pharm. J..

[84] Xu L., He W., Lu M., Yuan B., Zeng M., Tao G., Qin F., Chen J., Guan Y., He Z. (2018). Enzyme-assisted ultrasonic-microwave synergistic extraction and UPLC-QTOF-MS analysis of flavonoids from Chinese water chestnut peels. Ind. Crops Prod..

[85] Chen G., Li X., Saleri F., Guo M. (2016). Analysis of flavonoids in Rhamnus davurica and its antiproliferative activities. Molecules.

[86] Knittel D.N., Stintzing F.C., Kammerer D.R. (2014). Simultaneous determination of bufadienolides and phenolic compounds in sea squill (*Drimia maritima* (L.) Stearn) by HPLC-DAD-MS n as a means to differentiate individual plant parts and developmental stages. Anal. Bioanal. Chem..

[87] Ben Said R., Arafa I.H., Usam A.M., Abdullah Sulaiman A.-A., Kowalczyk M., Moldoch J., Oleszek W., Stochmal A. (2017). Tentative characterization of polyphenolic compounds in the male flowers of Phoenix dactylifera by liquid chromatography coupled with mass spectrometry and DFT. Int. J. Mol. Sci..

[88] Dehkordi F.J., Kharazian N., Lorigooini Z. (2020). Characterization of flavonoid components in *Scutellaria* L. species (Lamiaceae) using finger-printing analysis. Acta Biol. Cracoviensia Ser. Bot..

[89] Patyra A., Dudek M.K., Kiss A.K. (2022). LC-DAD–ESI-MS/MS and NMR Analysis of Conifer Wood Specialized Metabolites. Cells.

[90] Scalabrin E. (2015). Chemical Characterization of Plant Materials and Development of Analytical Methodologies for Metabolite Determination. Ph.D. Thesis.

[91] Huang G., Liang J., Chen X., Lin J., Wei J., Huang D., Zhou Y., Sun Z., Zhao L. (2020). Isolation and identification of chemical constituents from zhideke granules by ultra-performance liquid chromatography coupled with mass spectrometry. J. Anal. Methods Chem..

[92] Ağalar H., Ciftci G., Göger F., Kirimer N. (2018). Activity guided fractionation of Arum italicum miller tubers and the LC/MS-MS profiles. Rec. Nat. Prod..

[93] Demenciano S.d.C., Silva M.C.B.L.e., Alexandrino C.A.F., Kato Junior W.H., Figueiredo P.d.O., Garcez W.S., Campos R.P., Guimarães R.d.C.A., Sarmento U.C., Bogo D. (2020). Antiproliferative activity and antioxidant potential of extracts of *Garcinia gardneriana*. Molecules.

[94] Yao H., Chen B., Zhang Y., Ou H., Li Y., Li S., Shi P., Lin X. (2017). Analysis of the total biflavonoids extract from Selaginella doederleinii by HPLC-QTOF-MS and its in vitro and in vivo anticancer effects. Molecules.

[95] Miljić M., Rocchetti G., Krstić S., Mišan A., Brdar-Jokanović M., Marcheggiani F., Martinelli E., Lucini L., Damiani E. (2021). Comparative in vitro antioxidant capacity and terpenoid profiling of pumpkin fruit pulps from a Serbian *Cucurbita maxima* and *Cucurbita moschata* breeding collection. Antioxidants.

[96] Eklund P.C., Backman M.J., Kronberg L.Å., Smeds A.I., Sjöholm R.E. (2008). Identification of lignans by liquid chromatography-electrospray ionization ion-trap mass spectrometry. J. Mass Spectrom..

[97] Al Groshi A., Jasim H.A., Evans A.R., Ismail F.M., Dempster N.M., Nahar L., Sarker S.D. (2019). Growth inhibitory activity of biflavonoids and diterpenoids from the leaves of the Libyan Juniperus phoenicea against human cancer cells. Phytother. Res..

[98] Dong L.-B., He J., Wang Y.-Y., Wu X.-D., Deng X., Pan Z.-H., Xu G., Peng L.-Y., Zhao Y., Li Y. (2011). Terpenoids and norlignans from *Metasequoia glyptostroboides*. J. Nat. Prod..

[99] Miyazaki S., Kimura H., Natsume M., Asami T., Hayashi K.-i., Kawaide H., Nakajima M. (2015). Analysis of ent-kaurenoic acid by ultra-performance liquid chromatography-tandem mass spectrometry. Biochem. Biophys. Rep..

[100] Wang K., Bao L., Xiong W., Ma K., Han J., Wang W., Yin W., Liu H. (2015). Lanostane triterpenes from the Tibetan medicinal mushroom *Ganoderma leucocontextum* and their inhibitory effects on HMG-CoA reductase and α-glucosidase. J. Nat. Prod..

[101] You L.-P., Wang K.-X., Lin J.-C., Ren X.-Y., Wei Y., Li W.-X., Gao Y.-Q., Kong X.-N., Sun X.-H. (2023). Yin-chen Wu-ling powder alleviate cholestatic liver disease: Network pharmacological analysis and experimental validation. Gene.

[102] Pandey B., Baral R., Kaundinnyayana A., Panta S. (2023). Promising hepatoprotective agents from the natural sources: A study of scientific evidence. Egypt Liver J..

[103] Polimati H., Pragada R.R., Thuan N.H., Tatipamula V.B. (2022). Hepatoprotective potential of bioflavonoids. Stud. Nat. Prod. Chem..

[104] Li Y.L., Chen X., Niu S.Q., Zhou H.Y., Li Q.S. (2020). Protective Antioxidant Effects of Amentoflavone and Total Flavonoids from *Hedyotis diffusa* on H_2_O_2_-Induced HL-O_2_ Cells through ASK1/p38 MAPK Pathway. Chem. Biodivers..

[105] Shu-Mei Y., Wen-Yi K. (2011). Lowering blood lipid and hepatoprotective activity of amentoflavone from *Selaginella tamariscina* in vivo. J. Med. Plants Res..

[106] Huang Z.-Q., Chen P., Su W.-W., Wang Y.-G., Wu H., Peng W., Li P.-B. (2018). Antioxidant activity and hepatoprotective potential of quercetin 7-rhamnoside in vitro and in vivo. Molecules.

[107] Lee Y.-J., Beak S.-Y., Choi I., Sung J.-S. (2018). Quercetin and its metabolites protect hepatocytes against ethanol-induced oxidative stress by activation of Nrf2 and AP-1. Food Sci. Biotechnol..

[108] Alqasoumi S.I., Farraj A.I., Abdel-Kader M.S. (2013). Study of the hepatoprotective effect of *Juniperus phoenicea* constituents. Pak. J. Pharm. Sci..

[109] Ielciu I., Sevastre B., Olah N.-K., Turdean A., Chișe E., Marica R., Oniga I., Uifălean A., Sevastre-Berghian A.C., Niculae M. (2021). Evaluation of hepatoprotective activity and oxidative stress reduction of *Rosmarinus officinalis* L. shoots tincture in rats with experimentally induced hepatotoxicity. Molecules.

[110] Ibrahim N.A., El-Seedi H.R., Mohammed M.M.D. (2007). Phytochemical investigation and hepatoprotective activity of *Cupressus sempervirens* L. leaves growing in Egypt. Nat. Prod. Res..

[111] Salman A.A., El-Aleem A., Ibrahim M., Rahman A.-E., Ahmed A., Elhusseini T.S., El-Hadary A.A.E. (2017). Protective impacts of *Cupressus sempervirens* leaves extracts against paracetamol hepatotoxicity. Benha Vet. Med. J..

[112] Abdel-Kader M.S., Alanazi M.T., Saeedan A.S.B., Al-Saikhan F.I., Hamad A.M. (2017). Hepatoprotective and nephroprotective activities of *Juniperus sabina* L. aerial parts. J. Pharm. Pharmacogn. Res..

[113] Noor F., Tahir ul Qamar M., Ashfaq U.A., Albutti A., Alwashmi A.S., Aljasir M.A. (2022). Network pharmacology approach for medicinal plants: Review and assessment. Pharmaceuticals.

[114] Agarwal S., Mehrotra R. (2016). An overview of molecular docking. JSM Chem.

[115] Hassanein E.H., Mohamed W.R., Hussein R.M., Arafa E.-S.A. (2023). Edaravone alleviates methotrexate-induced testicular injury in rats: Implications on inflammation, steroidogenesis, and Akt/p53 signaling. Int. Immunopharmacol..

[116] Yuan H., Duan S., Guan T., Yuan X., Lin J., Hou S., Lai X., Huang S., Du X., Chen S. (2020). Vitexin protects against ethanol-induced liver injury through Sirt1/p53 signaling pathway. Eur. J. Pharmacol..

[117] Sun N., Yang T., Tang Y., Zhao Y., Wang H., Zhao S., Tan H., Li L., Fan H. (2022). Lycopene alleviates chronic stress-induced liver injury by inhibiting oxidative stress-mediated endoplasmic reticulum stress pathway apoptosis in rats. J. Agric. Food Chem..

[118] Liu F., Li Y., Yang Y., Li M., Du Y., Zhang Y., Wang J., Shi Y. (2021). Study on mechanism of matrine in treatment of COVID-19 combined with liver injury by network pharmacology and molecular docking technology. Drug Deliv..

[119] Drishya S., Dhanisha S.S., Guruvayoorappan C. (2022). Antioxidant-rich fraction of Amomum subulatum fruits mitigates experimental methotrexate-induced oxidative stress by regulating TNF-α, IL-1β, and IL-6 proinflammatory cytokines. J. Food Biochem..

[120] Tong C., Li J., Lin W., Cen W., Zhang W., Zhu Z., Lu B., Yu J. (2021). Inhibition of heat shock protein 90 alleviates cholestatic liver injury by decreasing IL-1β and IL-18 expression. Exp. Ther. Med..

[121] Mohagheghzadeh A., Badr P., Mohagheghzadeh A., Hemmati S. (2023). Hypericum perforatum L. and the Underlying Molecular Mechanisms for Its Choleretic, Cholagogue, and Regenerative Properties. Pharmaceuticals.

[122] Kömüves L.G., Feren A., Jones A.L., Fodor E. (2000). Expression of epidermal growth factor and its receptor in cirrhotic liver disease. J. Histochem. Cytochem..

[123] Liang D., Chen H., Zhao L., Zhang W., Hu J., Liu Z., Zhong P., Wang W., Wang J., Liang G. (2018). Inhibition of EGFR attenuates fibrosis and stellate cell activation in diet-induced model of nonalcoholic fatty liver disease. Biochim. Biophys. Acta (BBA)-Mol. Basis Dis..

[124] Fuchs B.C., Hoshida Y., Fujii T., Wei L., Yamada S., Lauwers G.Y., McGinn C.M., DePeralta D.K., Chen X., Kuroda T. (2014). Epidermal growth factor receptor inhibition attenuates liver fibrosis and development of hepatocellular carcinoma. Hepatology.

[125] Karabulut D., Ozturk E., Kuloglu N., Akin A.T., Kaymak E., Yakan B. (2020). Effects of vitamin B12 on methotrexate hepatotoxicity: Evaluation of receptor-interacting protein (RIP) kinase. Naunyn-Schmiedeberg’s Arch. Pharmacol..

[126] Morsy M.A., Abdel-Latif R., Hafez S., Kandeel M., Abdel-Gaber S.A. (2022). Paeonol Protects against Methotrexate Hepatotoxicity by Repressing Oxidative Stress, Inflammation, and Apoptosis-The Role of Drug Efflux Transporters. Pharmaceuticals.

[127] Cetin A., Kaynar L., Kocyigit I., Hacioglu S.K., Saraymen R., Ozturk A., Sari I., Sagdic O. (2008). Role of grape seed extract on methotrexate induced oxidative stress in rat liver. Am. J. Chin. Med..

[128] Abbas N.A.T., El-Sayed S.S., Abd El-Fatah S.S., Sarhan W.M., Abdelghany E.M.A., Sarhan O., Mahmoud S.S. (2023). Mechanistic aspects of ameliorative effects of Eicosapentanoic acid ethyl ester on methotrexate-evoked testiculopathy in rats. Naunyn-Schmiedeberg’s Arch. Pharmacol..

[129] Bu T., Wang C., Meng Q., Huo X., Sun H., Sun P., Zheng S., Ma X., Liu Z., Liu K. (2018). Hepatoprotective effect of rhein against methotrexate-induced liver toxicity. Eur. J. Pharmacol..

[130] Kalantar M., Kalantari H., Goudarzi M., Khorsandi L., Bakhit S., Kalantar H. (2019). Crocin ameliorates methotrexate-induced liver injury via inhibition of oxidative stress and inflammation in rats. Pharmacol. Rep..

[131] Hussain T., Tan B., Yin Y., Blachier F., Tossou M.C.B., Rahu N. (2016). Oxidative Stress and Inflammation: What Polyphenols Can Do for Us?. Oxidative Med. Cell. Longev..

[132] Koppelmann T., Pollak Y., Mogilner J., Bejar J., Coran A.G., Sukhotnik I. (2012). Dietary L-arginine supplementation reduces Methotrexate-induced intestinal mucosal injury in rat. BMC Gastroenterol..

