# Supplementary material for: Potential Hepatoprotective Effects of Chamaecyparis lawsoniana against Methotrexate-Induced Liver Injury: Integrated Phytochemical Profiling, Target Network Analysis, and Experimental Validation
Source: Antioxidants (Basel). 2023 Dec 14;12(12):2118. doi: 10.3390/antiox12122118 (PMC10740566; doi:10.3390/antiox12122118)
Supplement: Supplementary file 1 [file antioxidants-12-02118-s001.zip › Table S1.pdf]

Table S1. Target proteins, the corresponding grid coordinates, and amino acid residues of the active sites.

Target protein	PDB ID	Grid box coordinates		Amino acid residues of the active site
		Centers (x, y, z)	Sizes (x, y, z)	
TP53	8DC4	-3.38569, 44.2361, 70.4518	21.6746, 20.5837, 15.0543	Phe109, Leu145, Trp146, Val147, Pro151, Cys220, Glu221, Pro222, Pro223, Asp228, Ser229, Thr230 and Leu257
IL6	4NI9	-0.21833, 19.2354, 17.4484	15.4327, 15.8048, 16.1265	Leu33, Ile36, Ser37, Arg40, Thr43, His164, Leu167, Arg168 and Lys171
TNF-*α*	2AZ5	-21.1962, 75.6541, 32.956	16.589, 20.5662, 19.9422	HisA15, LeuA57, IleA58, TyrA59, SerA60, GlnA61, TyrA119, LeuA120, GlyA121, GlyA122, GlnA149, TyrA151, IleA155, LeuB57, TyrB59, SerB60, GlnB61, TyrB119, LeuB120, GlyB121 and TyrB151
HSP90AA1	8AGI	-25.8843, 19.7976, -14.2299	19.1259, 18.0473, 20.8229	Glu47, Leu48, Ser50, Asn51, Ser52, Asp54, Ala55, Lys58, Ile91, Val92, Asp93, Ile96, Gly97, Met98, Asp102, Asn106, Leu107, Ile110, Ala111, Gly132, Gly135, Val136, Gly137, Phe138, Tyr139, Val150, Thr184 and Val186
EGFR	7T4I	-1.80337, 21.6678, 52.6633	23.0255, 26.1052, 16.8854	Leu718, Gly719, Phe723, Val726, Ala743, Lys745, Leu747, Glu758, Ile759, Glu762, Cys775, Leu788, Thr790, Leu792, Met793, Gly796, Cys797, Asp837, Arg841, Asn842, Leu844, Thr854, Asp855, Gly857, Leu858 and Pro877
IL1B	1T4Q	-17.0683, 9.10508, -15.1524	16.9232, 17.5659, 16.972	Ser43, Ser45, Gly61, Leu62, Glu64, Lys65, Tyr68 and Pro91
BCL2	7LHB	23.8668, -3.05969, 23.1438	23.6987, 28.1142, 26.0363	Ala100, Asp103, Phe104, Asp111, Phe112, Glu114, Met115, Gln118, Val133, Glu136, Leu137, Trp144, Gly145, Val148, Ala149,

				Glu152, Phe153, Val156, Phe198, Tyr202
CASP3	3KJF	24.8943, -9.50017, 7.49641	22.7691, 19.6748, 18.5798	Trp206, Arg207, Asn208, Ser209, Lys210, Trp214, Glu248, Ser249, Phe250, Ser251 and Phe252
