# Supplementary material for: Potential Hepatoprotective Effects of Chamaecyparis lawsoniana against Methotrexate-Induced Liver Injury: Integrated Phytochemical Profiling, Target Network Analysis, and Experimental Validation
Source: Antioxidants (Basel). 2023 Dec 14;12(12):2118. doi: 10.3390/antiox12122118 (PMC10740566; doi:10.3390/antiox12122118)
Supplement: Supplementary file 1 [file antioxidants-12-02118-s001.zip › Table S3.pdf]

Table S3. Bioactive compounds of the ethanolic extract of *C. lawsoniana* aerial parts.

Compound	SMILES	Chemical structure
L-Threonic acid	C([C@@H]([C@H](C(=O)O)O)O)O	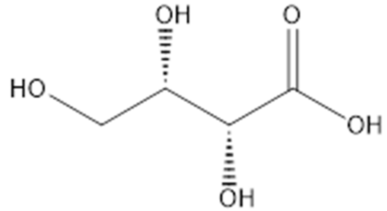
Vanillic acid glucoside	COC1=C(C=CC(=C1)C(=O)O)O[C@H]2[C@@H]([C@H]([C@@H]([C@H](O2)CO)O)O)O	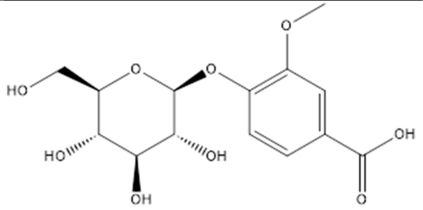
Quinic acid	C1[C@H](C([C@@H](CC1(C(=O)O)O)O)O)O	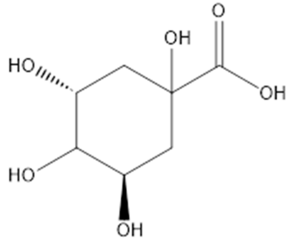
Caffeoylshikimic acid	C1[C@H]([C@@H]([C@@H](C=C1C(=O)[O-])O)O)OC(=O)/C=C/C2=CC(=C(C=C2)O)O	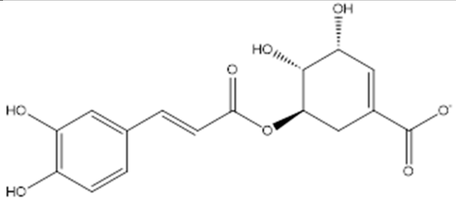

Ferulic acid *O*-glucoside	COC1=C(C=CC(=C1)C=CC(=O)O)OC2C(C(C(C(O2)CO)O)O)O	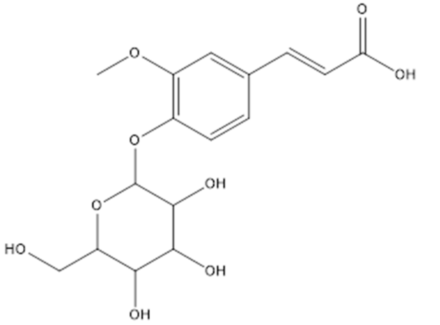
Coumaroylquinic acid	C1[C@H]([C@H]([C@@H](C[C@@]1(C(=O)O)O)OC(=O)/C=C/C2=CC=C(C=C2)O)O)O	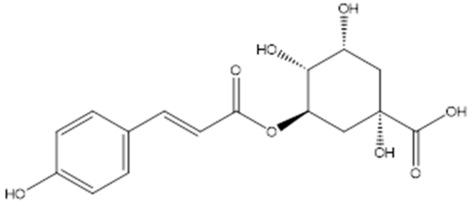
Roseoside	CC1=CC(=O)CC([C@]1(/C=C/[C@@H](C)O[C@H]2[C@@H]([C@H]([C@@H]([C@H](O2)CO)O)O)O)O)(C)C	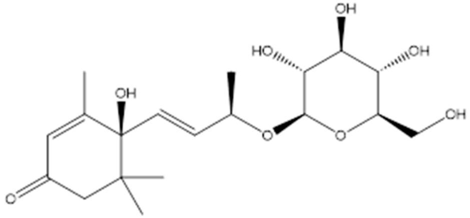
Sinapoyl D-glucoside	COC1=CC(=CC(=C1O)OC)/C=C/C(=O)OC2[C@@H]([C@H]([C@@H]([C@H](O2)CO)O)O)O	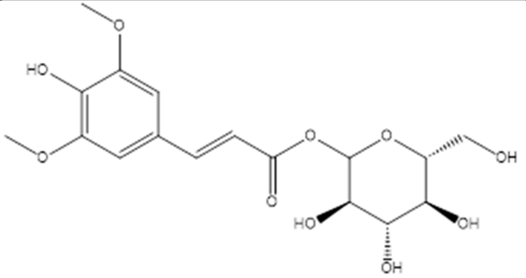

Pinopalustrin (Nortrachelogenin)	COC1=C(C=CC(=C1)C[C@H]2COC(=O)[C@@]2(CC3=CC(=C(C=C3)O)OC)O)O	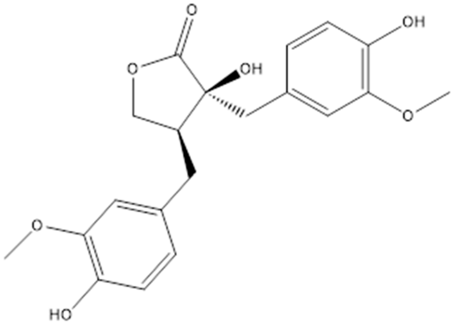
9,12,13-trihydroxyoctadeca-10,15-dienoic acid (Malyngic Acid)	CC/C=C\C[C@@H]([C@@H](/C=C/[C@H](CCCCCCCC(=O)O)O)O)O	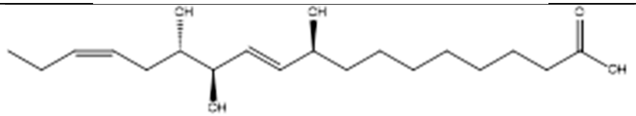
dihydrokaempferol (Aromadendrin)	C1=CC(=CC=C1[C@@H]2[C@H](C(=O)C3=C(C=C(C=C3O2)O)O)O)O	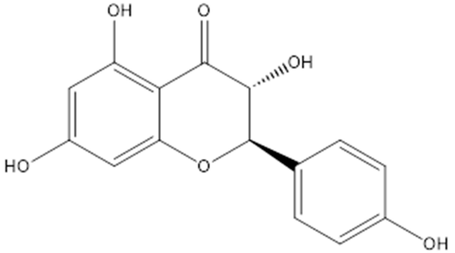
3,7-dimethylquercetin	COC1=CC(=C2C(=C1)OC(=C(C2=O)OC)C3=CC(=C(C=C3)O)O)O	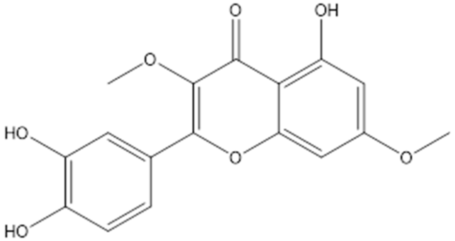
4',5,6,7-Tetramethoxyflavone (Scutellarein tetramethyl ether)	COC1=CC=C(C=C1)C2=CC(=O)C3=C(C(=C(C=C3O2)OC)OC)OC	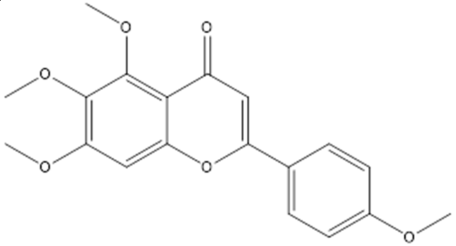

Phlorizin	C1=CC(=CC=C1CCC(=O)C2=C(C=C(C=C2O[C@H]3[C@@H]([C@H]([C@@H]([C@H](O3)CO)O)O)O)O)O)O	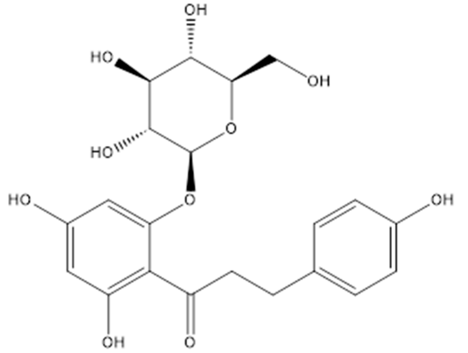
Kaempferol-3-*O*-arabinoside	C1[C@@H]([C@@H]([C@H]([C@@H](O1)OC2=C(OC3=CC(=CC(=C3C2=O)O)O)C4=CC=C(C=C4)O)O)O)O	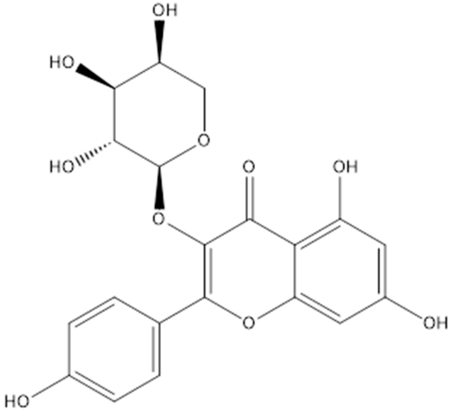
secoisolariciresinol guaiacylglyceryl ether	OC[C@H](CC1=CC=C(OC(CO)COC2=CC=CC=C2OC)C(OC)=C1)[C@@H](CC3=CC=C(O)C(OC)=C3)CO	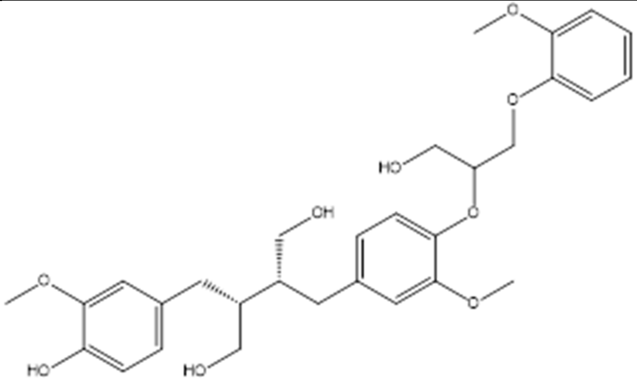

lariciresinol-4'-guaiacylglyceryl ether	COC1=CC(C[C@H]2CO[C@H](C3=CC=C(O)C(OC)=C3)[C@H]2CO)=CC=C1O[C@@H](CO)COC4=CC=CC=C4OC	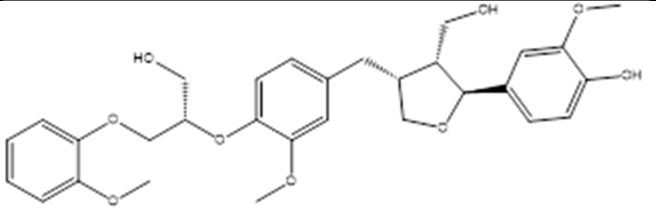
Naringenin	C1[C@H](OC2=CC(=CC(=C2C1=O)O)O)C3=CC=C(C=C3)O	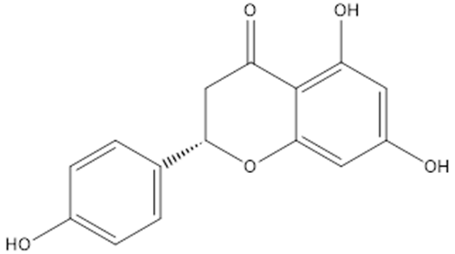
Protocatechualdehyde	C1=CC(=C(C=C1C=O)O)O	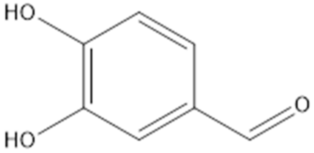
Matairesinol	COC1=C(C=CC(=C1)C[C@H]2COC(=O)[C@@H]2CC3=CC(=C(C=C3)O)OC)O	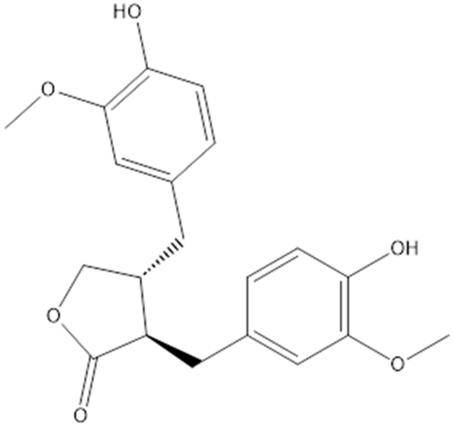

7-*O*-methylamentoflavone (Sequoiaflavone)	COC1=CC(=C2C(=C1)OC(=CC2=O)C3=CC(=C(C=C3)O)C4=C(C=C(C5=C4OC(=CC5=O)C6=CC=C(C=C6)O)O)O)O	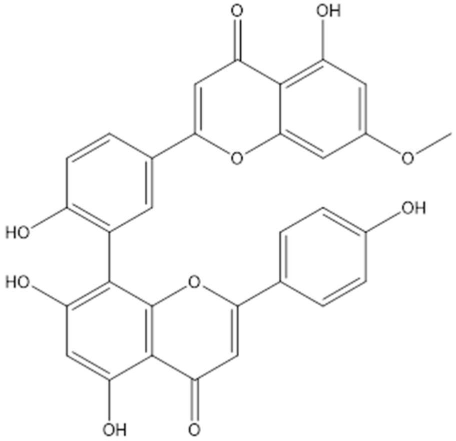
4\`-*O*-methylamentoflavone (Bilobetin)	COC1=C(C=C(C=C1)C2=CC(=O)C3=C(C=C(C=C3O2)O)O)C4=C(C=C(C5=C4OC(=CC5=O)C6=CC=C(C=C6)O)O)O	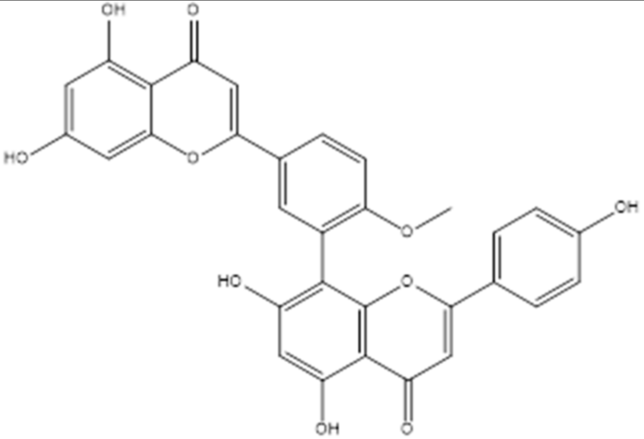
8alpha-8-Hydroxy-12-oxo-13-abieten-18-oic acid	CC(C)C1=CC2(CCC3C(C2CC1=O)(CCCC3(C)C(=O)O)C)O	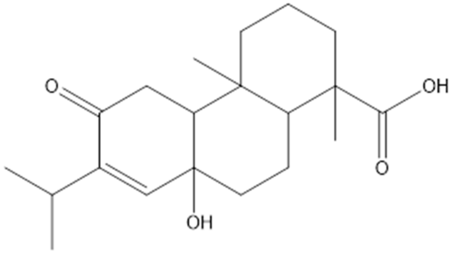

Copalic acid	C/C(=C\C(=O)O)/CC[C@@H]1C(=C)CC[C@H]2[C@]1(CCCC2(C)C)C	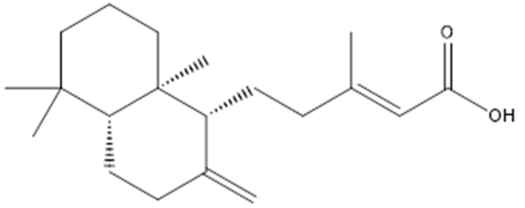
Isoginkgetin	COC1=CC=C(C=C1)C2=CC(=O)C3=C(O2)C(=C(C=C3O)O)C4=C(C=CC(=C4)C5=CC(=O)C6=C(C=C(C=C6O5)O)O)OC	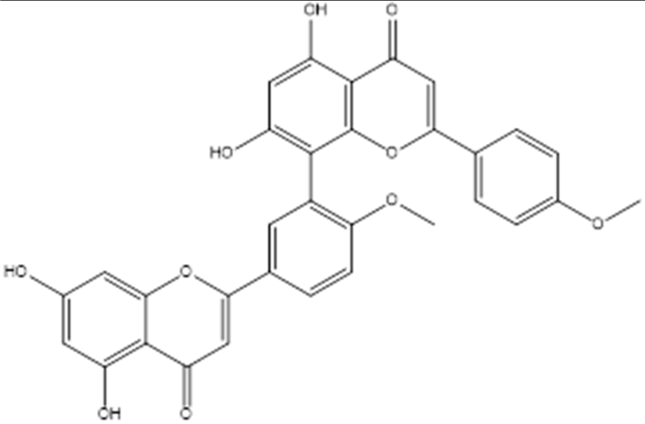
Robustaflavone 7,4`-dimethyl ether	COC1=C(C=C(C=C1)C2=CC(=O)C3=C(C=C(C=C3O2)OC)O)C4=C(C5=C(C=C4O)OC(=CC5=O)C6=CC=C(C=C6)O)O	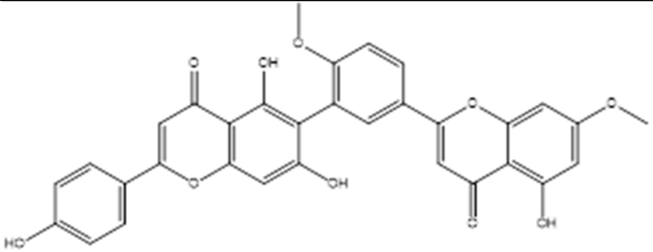

Cyclolariciresinol	COC1=C(C=C2[C@@H]([C@H]([C@@H](CC2=C1)CO)CO)C3=CC(=C(C=C3)O)OC)O	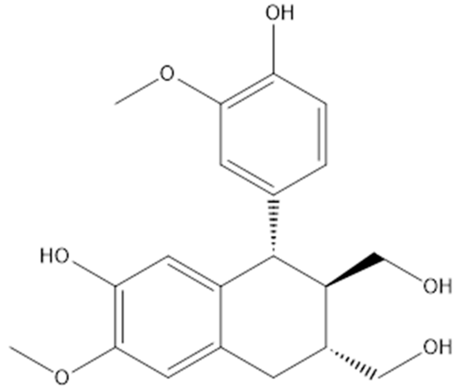
Carnosol	CC(C)C1=C(C(=C2C(=C1)[C@@H]3C[C@@H]4[C@@]2(CCCC4(C)C)C(=O)O3)O)O	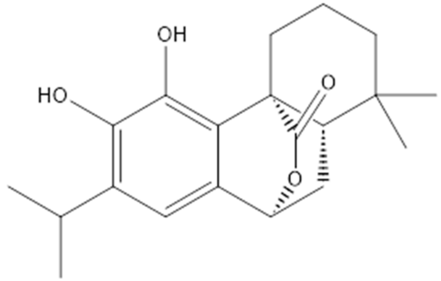
3-Hydroxysandaracopimaric acid	C[C@@]1(CC[C@H]2C(=C1)CC[C@@H]3[C@@]2(CC[C@@H]([C@@]3(C)C(=O)O)O)C)C=C	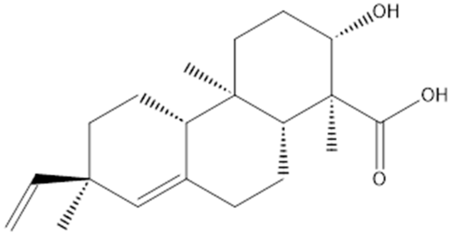
12alpha-hydroxy-8,15-isopimaradien-18-oic acid	C[C@]12CCC[C@@]([C@@H]1CCC3=C2C[C@@H]([C@](C3)(C)C=C)O)(C)C(=O)O	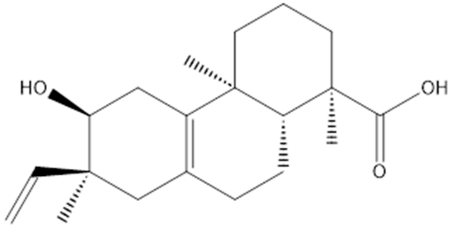

ent-kaurenoic acid	C[C@@]12CCC[C@@]([C@H]1CC[C@]34[C@H]2CC[C@H](C3)C(=C)C4)(C)C(=O)O	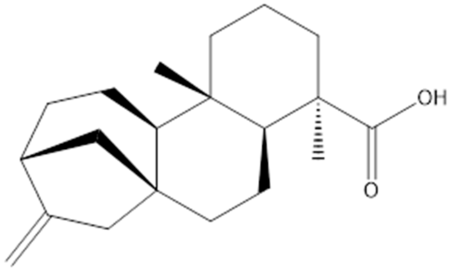
