# Supplementary figures and images for: Potential Hepatoprotective Effects of Chamaecyparis lawsoniana against Methotrexate-Induced Liver Injury: Integrated Phytochemical Profiling, Target Network Analysis, and Experimental Validation
Source: Antioxidants (Basel). 2023 Dec 14;12(12):2118. doi: 10.3390/antiox12122118 (PMC10740566; doi:10.3390/antiox12122118)

**Figure S1.** UPLC-ESI-MS/MS total ion chromatograms of CLAE in negative ion mode.

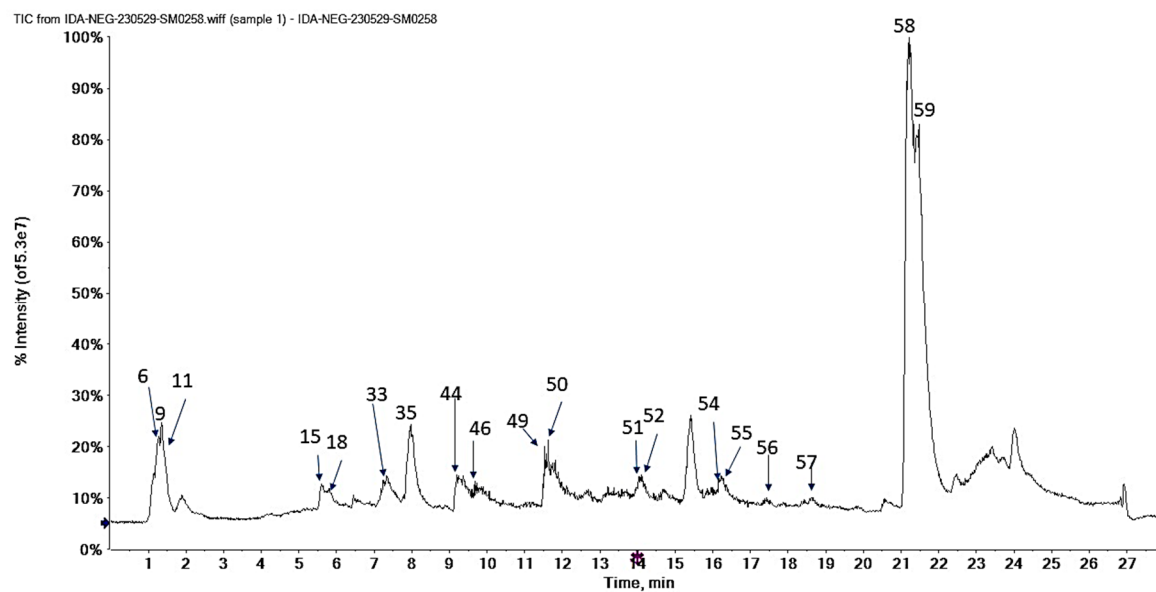

Supplement: Supplementary file 1 [file antioxidants-12-02118-s001.zip › Figure S1.pdf]

**Figure S2.** CLAE compounds-DILI targets network.

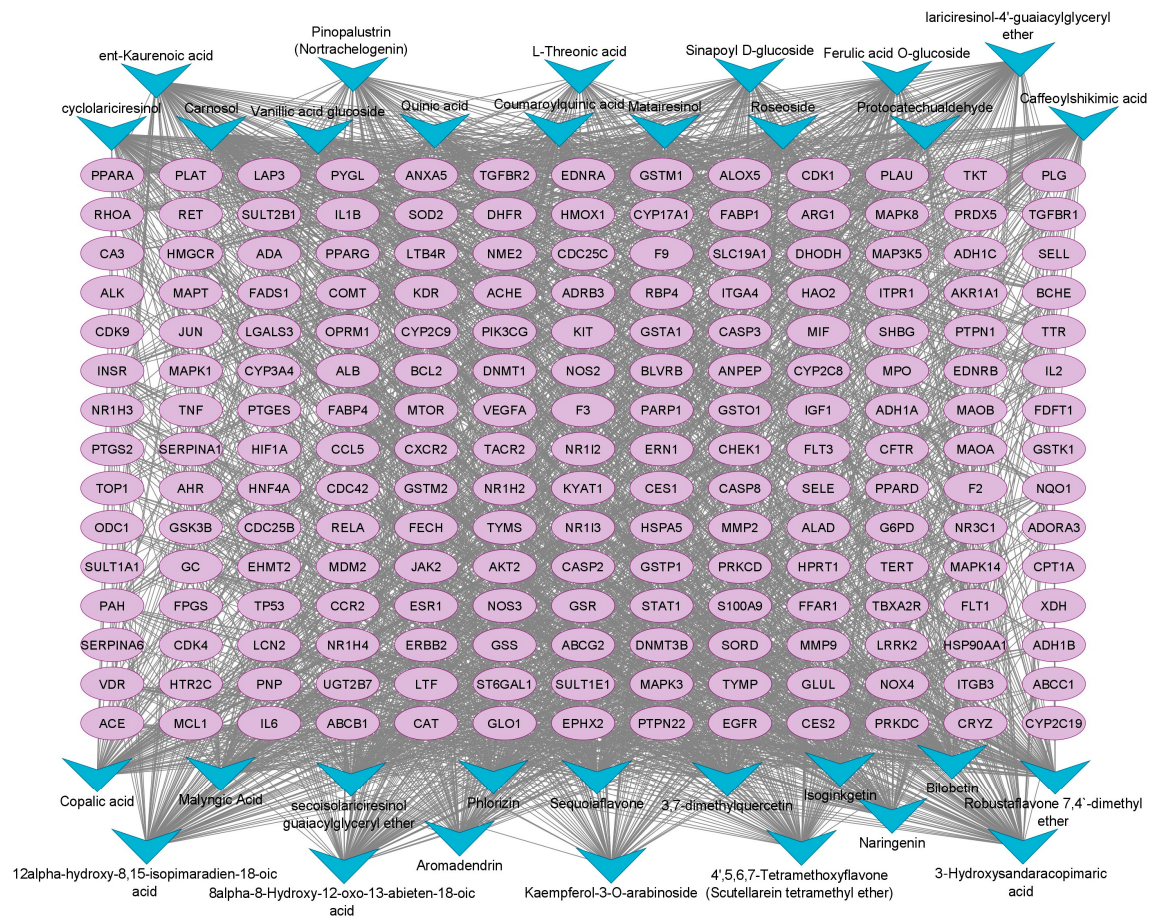

Supplement: Supplementary file 1 [file antioxidants-12-02118-s001.zip › Figure S2.pdf]
